# Detoxification of hostplant's chemical defence rather than its anti-predator co-option drives β-glucosidase-mediated lepidopteran counteradaptation

**DOI:** 10.1038/ncomms9525

**Published:** 2015-10-07

**Authors:** Spoorthi Poreddy, Sirsha Mitra, Matthias Schöttner, Jima Chandran, Bernd Schneider, Ian T. Baldwin, Pavan Kumar, Sagar S. Pandit

**Affiliations:** 1 Department of Molecular Ecology, Max-Planck-Institute for Chemical Ecology, 07745 Jena, Germany; 2 Department of Biosynthesis/NMR Research Group, Max-Planck-Institute for Chemical Ecology, 07745 Jena, Germany

## Abstract

The evolutionary plant–herbivore arms race sometimes gives rise to remarkably unique adaptation strategies. Here we report one such strategy in the lepidopteran herbivore *Manduca sexta* against its hostplant *Nicotiana attenuata*'s major phytotoxins, 17-hydroxygeranyllinalool diterpene glycoside, lyciumoside IV and its malonylated forms. We show that alkalinity of larval regurgitant non-enzymatically demalonylates the malonylated forms to lyciumoside IV. Lyciumoside IV is then detoxified in the midgut by *β-glucosidase 1*-catalysed deglycosylation, which is unusual, as typically the deglycosylation of glycosylated phytochemicals by insects results in the opposite: toxin activation. Suppression of deglucosylation by silencing larval *β-glucosidase 1* by plant-mediated RNAi causes moulting impairments and mortality. In the native habitat of *N. attenuata*, *β-glucosidase 1* silencing also increases larval unpalatability to native predatory spiders, suggesting that the defensive co-option of lyciumoside IV may be ecologically advantageous. We infer that *M. sexta* detoxifies this allelochemical to avoid its deleterious effects, rather than co-opting it against predators.

## Abstract (web summary)

During herbivory, deglycosylation of glycosylated phytochemicals may release aglycones which can be toxic when ingested. Here, Poreddy *et al*. show that ß-glucosidase in the tobacco hornworm midgut converts a toxic glycoside to a non-toxic form, a process which is crucial for larval development.

## Graphical abstract

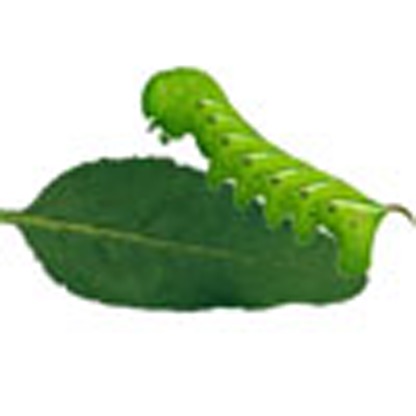

Plants commonly use lipophilic compounds such as cardenolides, iridoids, flavonoids, phenylpropanoids and several cyanogenic compounds as their major defences against their insect herbivores. These compounds are often glycosylated by the plants to increase their stability and to avoid self-intoxication; usually, such compounds are stored in specialized compartments to protect them from endogenous glycosidases^1^,^2^,^3^. Tissue damage resulting from insect feeding exposes these inactive pretoxins to their activating glycosidases resulting in the formation of toxic aglycones^3^,^4^,^5^. Released aglycones cause cellular damage in herbivores and severely affect their physiology and fitness^6^,^7^. Frequently, on ingestion, these glycosides are deglycosylated in the midgut by insect glycosidases^3^,^8^. To avoid such deglycosylation, some herbivores downregulate their glycosidases after ingesting glycosides^9^,^10^. Several herbivores have even evolved their own glycosylation systems to reglycosylate the formed aglycones^3^,^5^,^11^,^12^. Glycosides are more water soluble, have higher intracellular mobility and so are excreted faster than their aglycones^2^. Moreover, they are easily transported to vacuolar compartments and the apoplast by membrane-bound transporter systems, which often recognize their glycosyl residues^2^. Hence, deglycosylation or glycoside hydrolysis in insects is commonly thought to result in toxin activation and glycosylation is regarded as a form of detoxification^11^,^12^,^13^,^14^. Notably, most of this research has been conducted on compounds harbouring single sugar moieties; deglycosylation of phytochemicals containing more than one sugar moiety remains uninvestigated.

Several *Nicotiana* (Solanaceae) species produce 17-hydroxygeranyllinalool diterpene glycosides (HGL-DTGs) containing multiple sugar moieties^15^. HGL-DTGs have been reported to be biocidal and HGL-DTGs of *Nicotiana tabacum* retarded growth of *Heliothis virescens* larvae in artificial diet (AD) experiments^15^. The biosynthesis of several HGL-DTGs is induced in *N. attenuata* on attack by its specialist herbivore, *Manduca sexta* (*Ms*; Lepidoptera, Sphingidae). The susceptibility of *M. sexta* larvae to HGL-DTGs was demonstrated using different *Nicotiana* species such as *N. attenuata, N. bigelovii* and *N. clevelandii*, which differ in their HGL-DTG contents and composition, and, more convincingly, with different transgenic isogenic lines of *N. attenuata* with varying levels of HGL-DTGs^16^,^17^,^18^. *M. sexta* larvae grew three times larger than controls when they were fed transgenic *N. attenuata* plants depleted in their HGL-DTG content by silencing geranylgeranyl pyrophosphate synthase (*GGPPS*), the gene responsible for the synthesis of the HGL-DTG precursor, geranylgeranyl pyrophosphate^19^,^20^. In fact, HGL-DTGs were found to be more effective as chemical defences against *M. sexta* than some of *N. attenuata's* other well-characterized defence compounds, such as nicotine and trypsin proteinase inhibitors^19^.

*N. attenuata* HGL-DTGs are composed of an acyclic C_20_ HGL backbone decorated with glucose moieties at OH-3 and OH-17; glucose moieties can further be extended with either glucose or rhamnose moieties at OH-2′, OH-4′ or OH-6′ to produce a large diversity of structures. Frequently, malonyl groups are additionally attached to glucoses at OH-6′, resulting in additional structures^20^,^21^,^22^. Heiling *et al*.^20^ defined lyciumoside I as a precursor HGL-DTG, as it represents the first glycosylation of the HGL backbone at both the OH-3 and OH-17. HGL-DTGs having disaccharides attached to OH-3 and OH-17 are referred to as core HGL-DTGs. *N. attenuata* produces three core HGL-DTG structures namely, attenoside, nicotianoside III and lyciumoside IV (Lyc4). Core HGL-DTGs with malonyl groups on their sugar moieties are defined as malonylated HGL-DTGs and are further classified as singly or dimalonylated, based on the number of attached malonyl groups. Lyc4 with its singly malonylated form, nicotianoside I (Nic1), and its dimalonylated form, nicotianoside II (Nic2), constitutes ∼80% of the *N. attenuata*'s total HGL-DTG pool^20^.

Although HGL-DTGs severely affect larval growth, *M. sexta* larvae survive on the HGL-DTG-rich *N. attenuata*. We investigated *M. sexta*'s counteradaptation strategy against HGL-DTGs, using Lyc4 and its malonylated forms as model HGL-DTGs. We discovered unusual mechanisms evolved by *M. sexta* to demalonylate and then detoxify these allelochemicals. To reveal the significance of this atypical strategy, we applied a modern herbivore reverse genetics approach, plant-mediated RNA interference (PMRi)^23^,^24^. By silencing *M. sexta*'s Lyc4 ingestion-induced *β-glucosidase* (BG), we revealed that it is responsible for Lyc4 deglycosylation in the midgut and such detoxification is vital for larval development. Last, we used the PMRi in the native habitat of *N. attenuata*, the Great Basin Desert, Utah, USA to elucidate the role of Lyc4 detoxification in *M. sexta*'s interaction with its predators. We found that Lyc4 is a predator deterrent so avoiding its detoxification could benefit larvae that are vulnerable to predators. We discuss why *M. sexta* could have chosen the detoxification of Lyc4 in spite of it being ecologically disadvantageous.

## Results

### Nicotianoside I and II are demalonylated by the alkaline pH

**Figure 1:**
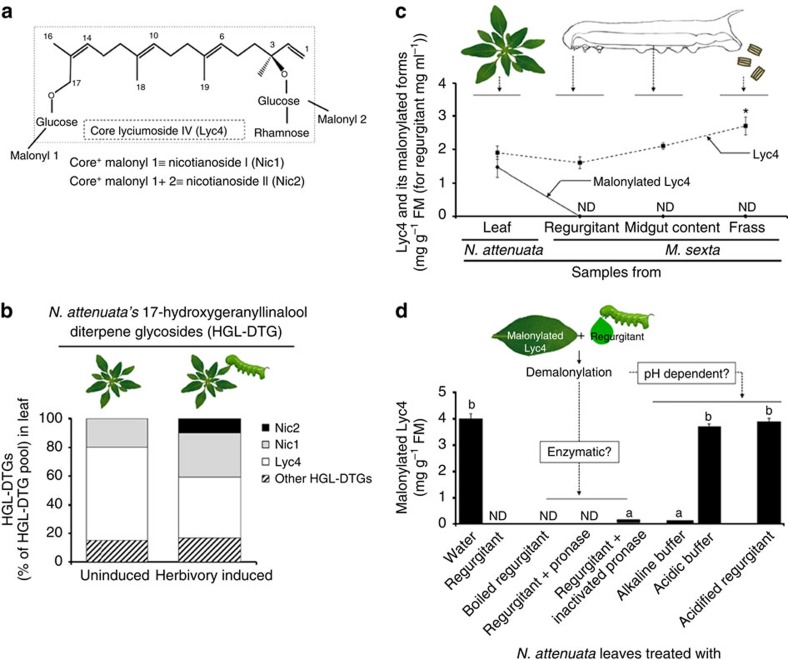
*N. attenuata'*s malonylated HGL-DTGs are demalonylated non-enzymatically in *M. sexta*'s alkaline regurgitant. (**a**) Structures of core lyciumoside IV (Lyc4) and its singly and dimalonylated forms, nicotianoside I (Nic1) and nicotianoside II (Nic2), respectively. (**b**) Proportions of Lyc4 and its malonylated forms Nic1 and Nic2 and other HGL-DTGs in uninduced and *M. sexta* herbivory-induced *N. attenuata* leaves showing that Lyc4 and its derivatives are the major HGL-DTGs (based on data from Heiling *et al*.^20^). (**c**) Profile of core and malonylated Lyc4 in *N. attenuata* leaf and in larval regurgitant, midgut content and frass showing that complete demalonylation occurs when the leaf comes into contact with larval regurgitant, at the time of ingestion by larvae (*F*_3,8_=19.81, *P*≤0.0001; significant differences (threshold: *P*≤0.05) between means (±s.e.) determined by Games Howell test (Welch's ANOVA); *n*=3). (**d**) Analysis to determine whether the demalonylation of Nic1 and Nic2 in larval regurgitant occurs enzymatically or by alkaline hydrolysis in the alkaline pH of the regurgitant; concentration of malonylated Lyc4 (Nic1 and Nic2) in *N. attenuata* leaf when it was crushed in water, regurgitant, boiled regurgitant, pronase-treated regurgitant, heat-inactivated pronase-treated regurgitant, alkaline buffer, acidic buffer and acidified regurgitant (*F*_4,20_=347.1, *P*≤0.001; significant differences (threshold: *P*≤0.05) between means (±s.e.) determined by Fisher's LSD test (one-way ANOVA); *n*=5). ND, not detected. ANOVA, analysis of variance; LSD, least significant difference.

Lyc4, Nic1 and Nic2 (Fig. 1a) constitute the major fraction of *N. attenuata'*s constitutive as well as herbivory-induced HGL-DTGs pool (Fig. 1b; Supplementary Fig. 1); therefore, we focused our study on these three compounds. To determine the fate of Nic1 and Nic2 in *M. sexta*, regurgitant (or oral secretion, as called by several researchers)^25^,^26^,^27^, midgut content and frass of larvae feeding on wild-type (WT) *N. attenuata* plants were analysed. Neither Nic1 nor Nic2 was detected in any of these samples. Absence of Nic1 and Nic2 in larval regurgitant suggested that demalonylation occurred in the regurgitant (Fig. 1c). To determine whether demalonylation resulted from the action of a plant or larval component, we incubated crushed *N. attenuata* leaves separately with either water or regurgitant in an *in vitro* demalonylation assay (Fig. 1d). Demalonylation occurred only in the sample incubated with regurgitant, whereas Nic1 and Nic2 were not found and the levels of Lyc4 increased. To test whether demalonylation was enzymatic, we incubated the leaf with enzyme-inactivated regurgitant; regurgitant was either boiled or treated with pronase to inactivate its enzymes. Complete demalonylation of Nic1 and Nic2 occurred in both treatments (Fig. 1d), suggesting that the reaction was non-enzymatic. Finally, to test whether demalonylation was pH dependent, we incubated the leaf with an alkaline buffer of pH-8.5 (because the regurgitant is alkaline^26^), an acidic buffer (pH-6) or acidified regurgitant (pH-6). Demalonylation occurred in the alkaline buffer but not in the acidic buffer and acidified regurgitant, confirming that ingested Nic1 and Nic2 were non-enzymatically demalonylated to Lyc4 by the alkalinity of larval regurgitant (Fig. 1d).

### *M. sexta* does not deglycosylate Lyc4 to form the aglycone

Usually, glycosides are deglycosylated by herbivores leading to a release of their toxic aglycones. Therefore, we first conducted a gas chromatography–mass spectrometry (GC-MS)-based analysis of foregut (*n*=4), midgut (*n*=4), hindgut (*n*=4), haemolymph (*n*=4), fat body along with skin (*n*=4) and frass (*n*=3) of larvae feeding on Lyc4-replete empty vector-transformed (EV) plants (a transformation control having similar levels of Lyc4, Nic1 and Nic2 to those in WT)^28^ to locate 17-HGL, the diterpene backbone of Lyc4, since only Lyc4 enters the larval gut because Nic1 and Nic2 are demalonylated to Lyc4 in the regurgitant. However, 17-HGL was not found in any of the above-mentioned samples. This led us to the U(H)PLC/ESI-QTOF-MS-based non-targeted Lyc4 metabolite search.

### Structure elucidation of a novel HGL-DTG from larval frass

**Figure 2:**
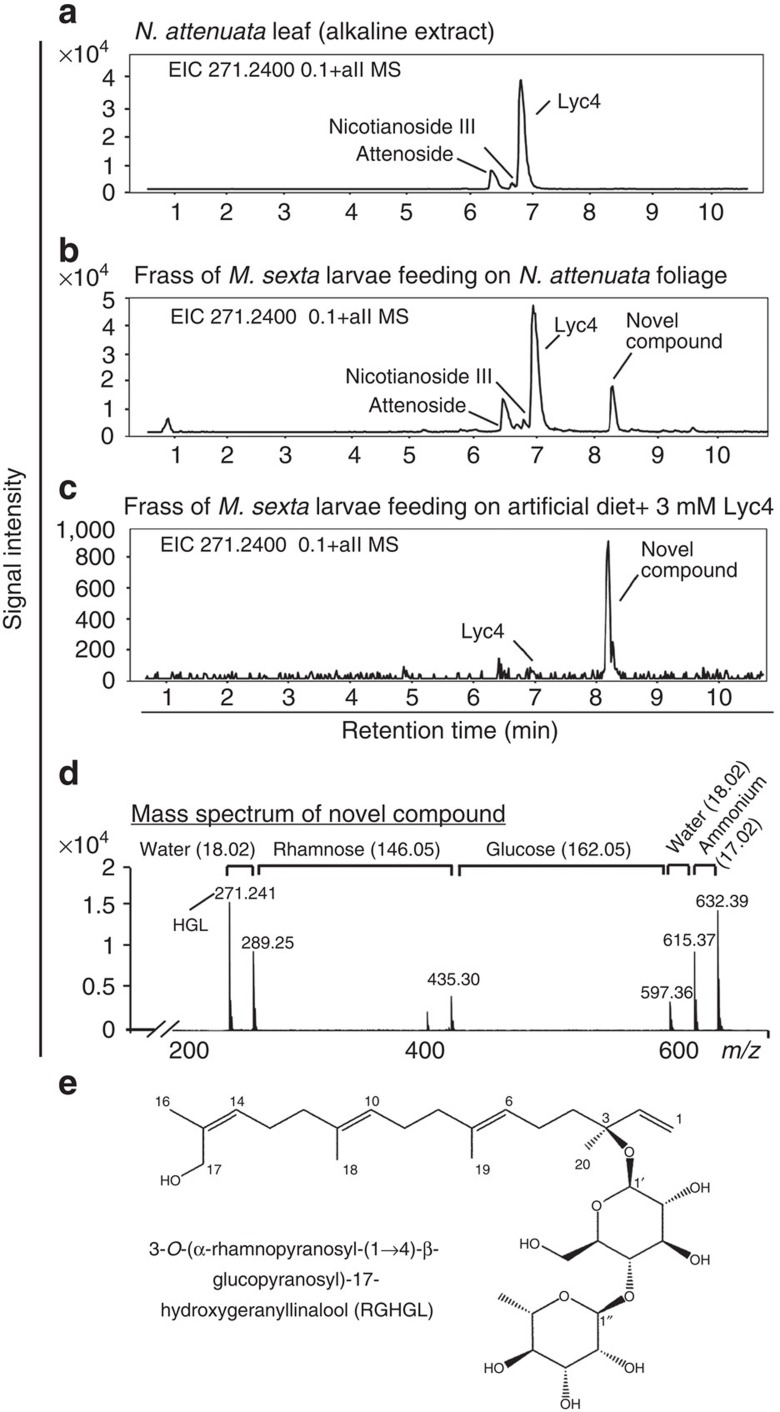
Lyc4 ingesting *M. sexta* larvae excrete a novel HGL-DTG. Ion (*m/z* 271.24≡HGL) extracted U(H)PLC/ESI-QTOF MS chromatograms showing *N. attenuata*'s major HGL-DTGs in (**a**) leaf (alkaline extract) and frass of larvae feeding on (**b**) *N. attenuata* foliage and (**c**) artificial diet (AD) containing 3 mM Lyc4 (physiological concentration of Lyc4 in an uninduced leaf). (**d**) Mass spectrum of the novel compound and prominent losses of functional groups. (**e**) Structure of the novel compound elucidated by NMR spectroscopy; compound was annotated as 3-*O*-α-rhamnopyranosyl-(1→4)-β-glucopyranosyl-17-hydroxygeranyllinalool (RGHGL).

To determine the fate of Lyc4 in the larval digestive system, we analysed the U(H)PLC/ ESI-QTOF-MS-generated HGL-DTG profile of the frass of larvae feeding on WT *N. attenuata* foliage and compared it with that of the alkaline buffer-treated WT leaf (Fig. 2a,b). An unknown compound having an HGL backbone, which was not found in leaf, was detected in the frass. This compound was also found in the frass of larvae fed Lyc4-supplemented AD, clearly indicating that it was a Lyc4 metabolite (Fig. 2c). The monoisotopic mass (*m/z*=614.37) of the compound and its fragmentation pattern were revealed by MS/MS analysis (Fig. 2d). The compound was purified from larval frass and its structure was elucidated by one-dimensional (^1^H and ^13^C NMR and APT) and two-dimensional (COSY, TOCSY, ROESY, HSQC and HMBC) NMR methods. The ^1^H NMR data of the novel compound resembled those reported for HGL-DTGs^20^,^29^,^30^. In the ^13^C NMR and APT spectra, 20 signals were detected for the aglycone, and 12 signals for two hexose units (Supplementary Table 1). The aglycone was identified as 17-hydroxygeranyllinalool^30^ as follows. The ^1^H NMR signals at δ 5.23 (1H, H-1a, ^3^*J*_H1a-H2_=17.8 Hz, ^2^*J*_H1a-H1b_=1.2 Hz), 5.20 (1H, H-1b, ^3^*J*_H1b-H2_=11.1 Hz, ^2^*J*_H1b-H1a_=1.2 Hz) and 5.93 (1H, H-2, ^3^*J*_H2-H1a_=17.8 Hz, ^3^*J*_H2-H1b_=11.1 Hz) indicated a terminal double bond. The signals at δ 5.11 (2H, *t*, *J*=7.3 Hz, H-6, H-10) and 5.26 (1H, *t, J*=7.1 Hz, H-14) were due to three protons at the tri-substituted double bonds. This was also demonstrated by HMBC correlations between the signals of H-6/H-10 with δ 16.2 (C-18/C-19), δ 40.9 (C-12) and δ 41.2 (C-8), respectively, and H-14 with δ 61.5 (C-17) and δ 21.7 (C-16). Vice versa, the isochronic methyl signals of H-18 and H-19 at δ 1.59 (6H, *s*), the signal of H-16 at δ 1.75 (3H, *s*) and their HMBC correlations with C-6 (δ 125.8), C-7 (δ 136.0), C-10 (δ 125.9), C-11 (δ 135.8), C-14 (δ 128.6) and C-15 (δ 135.7) confirmed the presence of three methyl groups at the olefinic carbons(Supplementary Figs 2–4). The methyl group at δ 1.38 (3H, *s*, H-20) attached to the quaternary C-3 exhibited HMBC correlations with C-4 (δ 42.8), C-3 (δ 81.6) and C-2 (δ 144.5) (Supplementary Fig. 5).

^1^H and ^13^C NMR signals at δ 4.36 (1H, *d*, *J*=8.0 Hz, H-1′)/δ 99.5 (C-1′) and 4.85 (1H, *d*, *J*=1.7 Hz, H-1")/δ 102.9 (C-1'') and further ^1^H NMR signals between δ 3.20 and 3.96 correlated by HSQC cross peaks to ^13^C signals characteristic of carbohydrates (δ∼70–80, Supplementary Table 1; Supplementary Fig. 6) confirmed the presence of two sugar moieties as already suggested by 12 corresponding signals in the ^13^C NMR spectrum. The coupling constants of ^3^*J*_H1′-H2′_=8.0 Hz (1H) and ^3^*J*_H1''-H2''_=1.7 Hz (H-1") indicated one sugar unit with β- and one with α-configuration, respectively, at the anomeric positions (Supplementary Table 1). The hydroxymethylene carbon signal at δ 62.2 (C-6′), together with the methyl signal at δ 18.0 (C-6"), the chemical shifts and the ^1^H–^1^H spin–spin coupling constants (Supplementary Table 1) were compatible with the presence of a β-glucose and an α-rhamnose in the molecule.

The differences between reported HGL-DTGs and the new compound were established as follows. The signal of C-17 at δ 61.5 appeared about 6.5 p.p.m. upfield of the C-17 signal reported for HGL-DTGs (δ∼68) bearing a carbohydrate unit at the 17-hydroxyl group. Therefore, it was concluded that in this compound, the 17-hydroxyl group remained unsubstituted and the two hexose units were attached to C-3 as a disaccharide. This was confirmed by the equivalent ^1^H NMR signal of the protons at C-17, which appeared as a singlet at δ 4.06 while the ^1^H NMR signals of H-17 of reported HGL-DTGs are inequivalent^30^,^31^. Attachment of the glucose to the 3-hydroxyl group of the diterpene backbone was verified by the HMBC correlation between H-1′ and C-3 (δ 81.6) (Supplementary Fig. 7). Interglycosidic HMBC correlations of H-1" with C-4′ (δ 79.7) and H-4′ with C-1" indicated 1→4 linkage between the rhamnose and the glucose units (Supplementary Fig. 7). From these data, the novel compound was identified as 3-*O*-[α-rhamnopyranosyl-(1→4)-β-glucopyranosyl]-17-hydroxygeranyllinalool (RGHGL; Fig. 2e).

Collected fractions of Lyc4 and RGHGL showed single peaks in U(H)PLC/ESI-QTOF-MS analysis indicating that the isolated compounds were pure. Relative quantification of Lyc4 and RGHGL was achieved using rebaudioside A as an internal standard for U(H)PLC/ESI-QTOF-MS-based measurements (Supplementary Fig. 8a–c) and as an external standard for high-performance liquid chromatography (HPLC; Agilent 1100 series)-based measurements (Supplementary Fig. 8d–f)^31^. Extraction efficiency of these compounds from the frass was >90% and it remained linear over a broad range of concentrations (0.02–0.32 μg mg^−1^; Supplementary Fig. 8g–i).

### Lyc4 ingestion induces BG1 in the larval midgut

**Figure 3:**
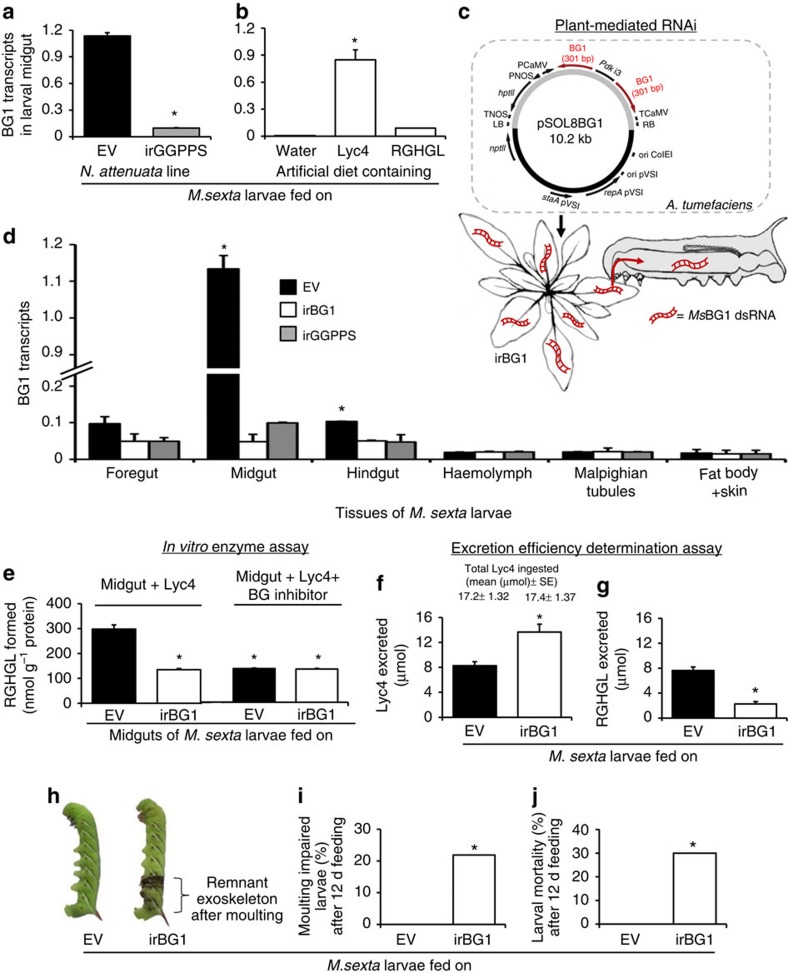
Silencing of Lyc4 ingestion-induced, midgut-expressed *BG1* impairs larval Lyc4 deglucosylation, moulting and survival. BG1 transcripts (relative to ubiquitin) in midguts of fourth-instar larvae feeding on (**a**) Lyc4-containing EV and Lyc4-depleted irGGPPS plants (*F*_1,10_=833.5, *P*≤0.0001; significant difference (*P*≤0.05) between means (±s.e.) determined by Fisher's LSD test (one-way ANOVA); *n*=6) and (**b**) AD containing water (control), 6 mM Lyc4 or 6 mM RGHGL (*F*_2,15_=5812.13, *P*≤0.0001; significant differences (*P*≤0.05) between means (±s.e.) determined by Games Howell test (Welch's ANOVA); *n*=6). (**c**) Plant-mediated RNAi: pSOL8 binary vector constructed to express 301 bp *Ms*BG1 dsRNA in *N. attenuata* and the trophic transfer of dsRNA from plant to larvae. (**d**) BG1 transcripts (relative to ubiquitin) in various tissues of fourth-instar larvae feeding on EV, irBG1 and irGGPPS plants (midgut: *F*_2,15_=9.53 *P*≤0.002; hindgut: *F*_2,15_=248, *P*≤0.0001; significant differences (*P*≤0.05) between means (±s.e.) determined by Games Howell test (Welch's ANOVA, separately conducted for each tissue); *n*=6 in all bars). (**e**) RGHGL formed *in vitro* enzyme assays containing midgut extracts of fourth-instar EV- and irBG1-feeding larvae and either (1 mM) Lyc4 (*F*_3,8_=104.9, *P*≤0.0001; significant differences (*P*≤0.05) between means (±s.e.) determined by Fisher's LSD test (one-way ANOVA); *n*=3) or 1 mM Lyc4+1 mM BG inhibitor, δ-gluconolactone (*n*=3). Excretion (% of total Lyc4 ingested) of (**f**) Lyc4 (*F*_1, 25_=13.95, *P*≤0.001; significant difference (*P*≤0.05) between means (±s.e.) determined by Fisher's LSD test (one-way ANOVA); *n*=13 (EV) and 14 (irBG1)) and (**g**) RGHGL (*F*_1, 25_=62.65, *P*≤0.0001; significant difference (*P*≤0.05) between means (±s.e.) determined by Fisher's LSD test (one-way ANOVA); *n*=13 (EV) and 14 (irBG1)) by fourth-instar EV- or irBG1-feeding larvae. (**h**) Phenotype (in irBG1-feeding larva) and (**i**) percentage of moulting impairment (significant difference (*P*≤0.05) determined by Fisher's exact test of frequencies; *n*=30) and (**j**) mortality (%) in larvae feeding (for 12 d) on EV or irBG1 plants (significant difference (*P*≤0.05) determined by Fisher's exact test of frequencies; *n*=30). ANOVA, analysis of variance; d, days; LSD, least significant difference.

NMR analysis showed that Lyc4 and RGHGL structures were similar, except that the C-17 of RGHGL lacked a glucose moiety (Fig. 2e), and since such deglycosylation reactions are often catalysed by BGs^4^, we searched for the BG involved in RGHGL formation. We identified three *BG*s (*BG1*, *BG2* and *BG3* NCBI accession no: FK816842, FK816724 and FK816837, respectively) from the collection of *M. sexta glycoside hydrolases* (Supplementary Fig. 9a). Transcript abundance of *BG1* (Fig. 3a) but not *BG2* and *BG3* (Supplementary Fig. 9b,d) was higher in midguts of larvae feeding on EV plants than in the larvae feeding on Lyc4-depleted inverted repeat (ir)GGPPS plants. BG1 transcript accumulation was also increased in the midguts of larvae feeding on AD containing 6 mM Lyc4 but not containing 6 mM RGHGL (Fig. 3b), suggesting that *BG1* was induced in response to the ingestion of Lyc4 and not involved in the downstream metabolism of RGHGL; this was supported by the fact that when larvae (*n*=6) were fed RGHGL in AD, they excreted no other compound having HGL backbone than RGHGL. The abundance of BG2 and BG3 transcripts in the midgut was not elicited in response to Lyc4 and RGHGL ingestions (Supplementary Fig. 9c,e).

### Silencing of *MsBG1* by PMRi

To understand *BG1*'s role in larval Lyc4 metabolism and its impact on larval growth and development, we silenced the expression of *BG1* in larval midguts, using PMRi (Fig. 3c). Stable transgenic irBG1 lines of *N. attenuata* expressing 301 bp *Ms*BG1 double-stranded RNA (dsRNA) were generated using *Agrobacterium tumefaciens*-mediated transformation of an ir of 301 bp *Ms*BG1 complementary DNA (cDNA) fragment. Single transgene insertions in two independently transformed irBG1 lines (375-10 and 379-8) were confirmed by Southern blot hybridization (Supplementary Fig. 10a). Presence of small dsRNA (21–24 nt) in irBG1 leaves and in midguts of irBG1-feeding larvae was confirmed by Northern blot hybridization (Supplementary Fig. 10b); BG1 dsRNA was not detected in EV leaves and in midguts of EV-feeding larvae (controls). Growth, morphology and levels of Lyc4 and other secondary metabolites (nicotine, rutin, caffeoyl putrescine and chlorogenic acid) of both irBG1 lines were similar to those of EV plants (Supplementary Fig. 10d,e). Midguts of larvae feeding on irBG1 plants showed >90% lower abundance of BG1transcripts than that of EV-feeding larvae (Fig. 3d). BG1 transcript abundance was also reduced in foregut and hindgut of irBG1-feeding larvae (Fig. 3d). Efficiencies of silencing by both irBG1 lines 375-10 and 379-8 were similar; therefore, irBG1 (375-10) was randomly selected for further experiments. To determine whether *BG1* silencing was specific and had no off-target effects on the expressions of other similar genes, we quantified the transcripts of closely related *BG2* and *BG3* (60.2% and 51.5% nucleotide similarity to *BG1*, respectively). Transcript abundance of *BG2* and *BG3* in the midguts of larvae feeding on irBG1 plants were similar to those of larvae feeding on EV plants (Supplementary Fig. 10f,g), suggesting that the *BG1* silencing by PMRi was sequence specific. Hereafter, we refer the irBG1-feeding larvae as ‘BG1-silenced' larvae.

### *MsBG1* silencing impairs larval Lyc4 metabolism

The influence of *BG1* silencing on larval Lyc4 metabolism was tested using an *in vitro* Lyc4 deglycosylation assay with the midgut tissue extracts of BG1-silenced and control larvae. The amount of RGHGL formed by the midguts of BG1-silenced larvae was significantly lower (55%) than that formed by the midguts of EV-fed larvae (Fig. 3e); similarly reduced RGHGL formation was observed when midguts of control and BG1-silenced larvae were incubated with Lyc4 and a BG inhibitor, δ-gluconolactone (Fig. 3e). These results revealed that Lyc4 deglycosylation activity was impaired in the irBG1-feeding larvae.

Further, to test the effect of *BG1* silencing on larval Lyc4 accumulation, we quantified the amounts of Lyc4 and RGHGL in foregut, midgut, hindgut tissues, haemolymph, Malpighian tubules and fat body with skin of fourth-instar BG1-silenced larvae. These larvae showed significantly higher Lyc4 content in their midgut, hindgut, haemolymph and fat body, and lower RGHGL content in midgut compared with those of EV-fed larvae (Supplementary Fig. 11a,b). Excretion efficiency determination assays^32^,^33^ revealed that although the amounts of Lyc4 ingested by the BG1-silenced and EV-fed larvae were similar, BG1-silenced larvae excreted 40% more Lyc4 and 70% less RGHGL in their frass than did EV-fed larvae (Fig. 3f,g). Before determining these amounts of excreted Lyc4 and RGHGL, we quantified the accuracy of our extraction procedure and also determined that these compounds were stable in larval frass over the 24-h period of the assay. For these examinations, we spiked each compound (separately) into fresh frass of irGGPPS-fed larvae, incubated them for 0 h (control) and 24 h under the assay conditions, extracted the spiked compounds and quantified them. This analysis revealed that the efficiencies of extraction of Lyc4 and RGHGL from frass were 88% and 91%, respectively, and that these compounds were stable in frass over the assay period (Supplementary Fig. 11c).

### Suppressed Lyc4 metabolism causes larval moulting impairment

**Figure 4:**
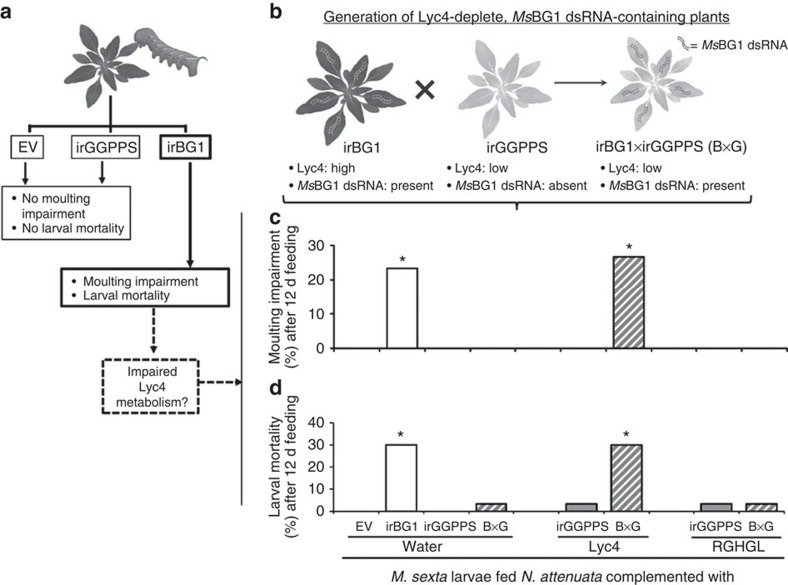
Moulting impairment and mortality in BG1-silenced larvae is associated with suppressed Lyc4 metabolism. (**a**) Schematic showing the motivation for the hypothesis that suppressed Lyc4 metabolism in irBG1-feeding larvae causes moulting impairment and mortality. (**b**) Generation of *Ms*BG1-containing and Lyc4-depleted transgenic *N. attenuata* plants (B × G) by crossing irBG1 with irGGPPS plants. (**c**) Moulting impairment (%) (significant difference (*P*≤0.05) determined by Fisher's exact test of frequencies; *n*=30) and (**d**) mortality (%) (significant difference (*P*≤0.05) determined by Fisher's exact test of frequencies; *n*=30) in larvae after 12 d feeding on water coated EV, irBG1, irGGPPS and B × G leaves, Lyc4 coated (final concentration 6 mM) irGGPPS and B × G leaves and RGHGL-coated (final concentration 6 mM) irGGPPS and B × G leaves. d, days.

*BG1* silencing had no effect on larval growth, however, during every moult (until the fourth-instar) we found that some BG1-silenced larvae were unable to shed their exoskeleton; collectively, 21.8% BG1-silenced larvae (*n*=32) failed to moult during the first three instar changes (Fig. 3h). Hereafter, we refer to this phenomenon as ‘moulting impairment'. Larvae unable to free themselves from the old exoskeleton stopped feeding, gradually melanized and died within 48 h of (failed) moulting (Fig. 3i,j; Supplementary Fig. 11d); moulting impairment and elevated mortality were not observed in the EV-fed larvae (Fig. 3i,j). Some BG1-silenced larvae (∼10% of the total dead) died soon after moulting but did not show the unshed exoskeleton or melanization phenotypes; these were not counted as moulting-impaired larvae. Although it was demonstrated that *BG1* silencing suppressed Lyc4 deglucosylation, whether moulting impairment was caused by *BG1* silencing or by Lyc4 metabolism suppression remained unclear (Fig. 4a). Along with the BG1 dsRNA, irBG1 plants contained Lyc4 levels equal to those of EV plants so the effects of *BG1* silencing and Lyc4 deglucosylation suppression could not be uncoupled in the larvae feeding on these plants. Therefore, we generated Lyc4-depleted and *Ms*BG1 dsRNA-containing plants by crossing irBG1 with irGGPPS plants (irBG1 × irGGPPS; hereafter called B × G) (Fig. 4b). Levels of HGL-DTGs of B × G plants were similar to the low levels of irGGPPS plants. Although *BG1* silencing in B × G-fed larvae was similar to that of irBG1-fed larvae (Supplementary Fig. 12a), moulting impairment and resulting mortality were not observed (Fig. 4c,d). Levels of Lyc4 and RGHGL in the bodies (without gut contents) of B × G-fed larvae were similar to those in the bodies of irGGPPS-fed larvae (Supplementary Fig. 12b,c). This indicated that the moulting impairment phenotype did not result from the effect of *BG1* silencing on larval primary metabolism but was related to Lyc4 deglucosylation by BG1. To test this, we complemented B × G and irGGPPS leaves with Lyc4 and RGHGL by topical coating; water-coated EV, irBG1, irGGPPS and B × G leaves were used as controls. More than 90% of topically coated Lyc4 and RGHGL could be recovered from coated leaves after 24 h, indicating that these compounds were stable on leaves during larval feeding (Supplementary Fig. 12d). Larvae feeding on Lyc4-coated B × G plants (*n*=30) showed high moulting impairment (22%) and mortality (28%) as did the larvae feeding on water-coated irBG1 leaves (Fig. 4c,d). Larvae feeding on RGHGL- or water-coated B × G and irGGPPS leaves and water-coated EV leaves (*n*=30) did not show these phenotypes (Fig. 4c,d).

Moulting impairment and mortality associated with the Lyc4 ingestion by BG1-silenced larvae were not observed after the ingestion of Lyc4 or RGHGL alone or after silencing *BG1* in the absence of Lyc4 ingestion. This clearly showed that the *BG1* function is vital for larvae ingesting large quantities of Lyc4 produced by *N. attenuata*.

### A native predator fails to ingest the BG1-silenced larvae

**Figure 5:**
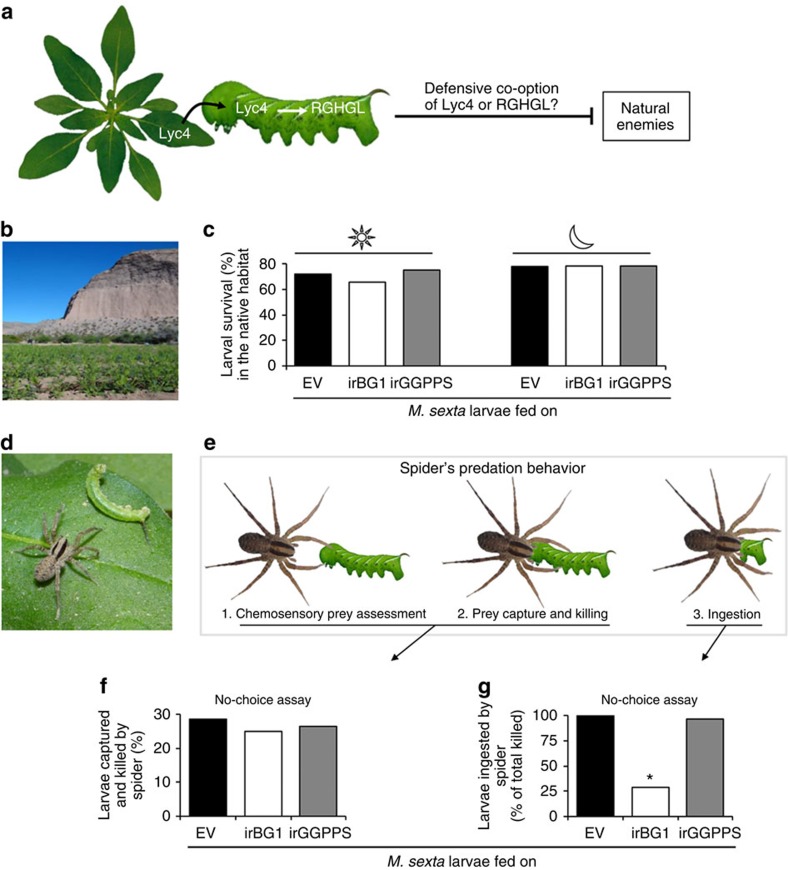
In the native habitat, spiders capture and kill but do not ingest the BG1-silenced *M. sexta* larvae. To test (**a**) whether Lyc4 or RGHGL is defensively co-opted by larvae against the natural enemies in the native habitat, (**b**) EV, irBG1 and irGGPPS plants were grown in the Great Basin Desert, Utah. (**c**) Diurnal and nocturnal survival (%) of larvae feeding on these plants (*n*=32 larvae per line). (**d**) Native *C. parallela* spider attacking *M. sexta* larva. (**e**) Three stages of spider's predation behaviour. Spider's (**f**) prey capture and killing (%; *n*=28) and (**g**) prey ingestion (% of total killed; significant difference (*P*≤0.05) determined by Fisher's exact test of frequencies; *n*=28) in a no-choice assay (1 h) on second-instar *M. sexta* larvae feeding on EV and irBG1 and irGGPPS plants.

Several herbivores defensively co-opt ingested plant metabolites either with or without metabolism^3^,^11^,^34^,^35^,^36^; so we hypothesized that *M. sexta* larvae co-opt Lyc4 or RGHGL to use against their natural enemies (Fig. 5a). To test this, we analysed the effect of *BG1* silencing on larval survival in the host's predator-teemed native habitat^32^. We introduced EV, irBG1 and irGGPPS stable transgenic lines into a field plot in the Great Basin Desert, Utah (Fig. 5b) and allowed *M. sexta* larvae to feed on these plants. Diurnal and nocturnal survivorships of BG1-silenced larvae (68% and 69%, respectively) did not differ significantly from those of larvae feeding on EV (70% and 70%, respectively) and irGGPPS (71% and 70%, respectively) plants (*n*=32 larvae per line, in both the experiments; Fig. 5c). However, while recording nocturnal survivorship, we found some carcasses of partially eaten larvae on irBG1 plants. Since the moulting-impaired BG1-silenced larvae were not used in these assays, occurrence of such carcasses was likely not related to moulting impairment. Hence, that such carcasses were not found on EV or irGGPPS plants, clearly suggested that the BG1-silenced larvae experienced some distinctive predator behaviour. During the night, *M. sexta* larvae are mainly preyed on by the wolf spiders (*Camptocosa parallela*; Lycosidae), which are abundant in this habitat^32^,^37^ (Fig. 5d). Therefore, we hypothesized that these spiders were responsible for the carcasses of partially eaten larvae. To test this, we conducted choice and no-choice predation bioassays using these spiders. During these assays we closely observed spider's predation behaviour, which generally consists of three steps (1) prey assessment by tapping it with chemosensory-endowed legs and palps, (2) prey capture followed by killing and (3) ingestion of the prey (Fig. 5e). In choice assays, we offered two larvae (one each from EV fed and irBG1 fed or EV fed and irGGPPS fed or irBG1 fed and irGGPPS fed) to each spider (*n*=30). Spiders did not show any preference for the given larvae, suggesting that they were unable to differentiate between them (Supplementary Fig. 13a–c). In no-choice assays, each spider was offered only one EV-, irBG1- or irGGPPS-fed larva (*n*=30). Frequencies of prey capture and killing were similar for EV-, irBG1- and irGGPPS-fed larvae. We observed that all the killed EV- and irGGPPS-fed larvae were completely eaten; however, spiders did not ingest ∼75% of the BG1-silenced larvae that they killed (Fig. 5f,g). These results are consistent with the hypothesis that spiders were responsible for the carcasses of partially eaten larvae we found during the nocturnal survivorship assays.

### High Lyc4 concentration hinders spider's prey ingestion

**Figure 6:**
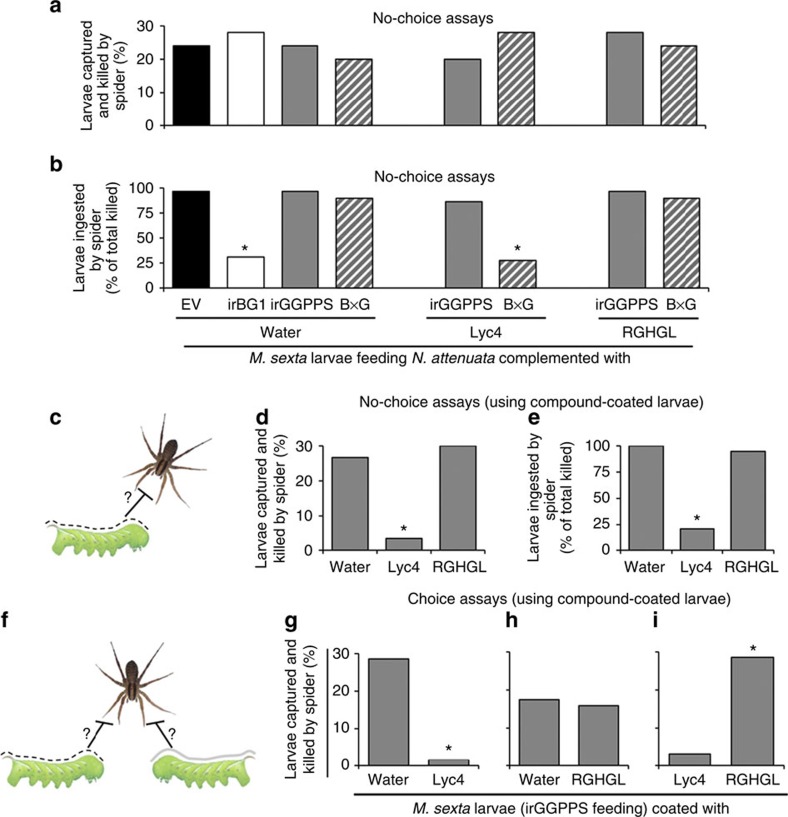
High larval body Lyc4 concentration hinders spiders' prey ingestion and topically coated Lyc4 deters spiders. Spider's (**a**) prey capture and killing (%; *n*=25) and (**b**) prey ingestion (% of total killed; significant difference (*P≤*0.05) determined by Fisher's exact test of frequencies; *n*=25) in a no-choice assay (1 h) using second-instar *M. sexta* larvae feeding on EV and irBG1 leaves coated with water (control) and irGGPPS and B × G leaves coated with water (control), Lyc4 (final concentration 6 mM) and RGHGL (final concentration 6 mM). (**c**) Schematic of a no-choice assay. (**d**) Spider's prey capture and killing (%; significant difference (*P*≤0.05) determined by Fisher's exact test of frequencies; *n*=30) and (**e**) prey ingestion (% of total killed; significant difference (*P*≤0.05) determined by Fisher's exact test of frequencies; *n*=30) in no-choice assays (1 h) using second-instar water-, Lyc4- or RGHGL-coated (final concentration 6 mM for both Lyc4 and RGHGL) *M. sexta* larvae feeding on irGGPPS plants and (**f**) schematic of a choice assay. Spider's prey capture and killing (%) in choice assays (1 h) using irGGPPS-feeding second-instar *M. sexta* larvae coated with (**g**) water or Lyc4 (final concentration 6 mM; significant difference (*P*≤0.05) determined by Fisher's exact test of frequencies; *n*=21), (**h**) water or RGHGL (final concentration 6 mM; *n*=21) and (**i**) Lyc4 (final concentration 6 mM) or RGHGL (final concentration 6 mM; significant difference (*P*≤0.05) determined by Fisher's exact test of frequencies; *n*=21).

To investigate whether Lyc4 or RGHGL content of BG1-silenced larvae prevented spiders from ingesting them, we conducted no-choice assays. We offered each spider a larva fed on water-coated EV, irBG1, irGGPPS or B × G leaves, Lyc4-coated irGGPPS or B × G leaves or RGHGL-coated irGGPPS or B × G leaves. Prey capture and kill frequencies were similar for all these larvae (*n*=25 in each treatment) (Fig. 6a). However, prey ingestion frequencies were reduced in the cases of larvae fed on water-coated irBG1 and Lyc4-coated B × G leaves (Fig. 6b); spiders preying on these larvae, but not EV- and irGGPPS-fed larvae, showed signs of distress after an unsuccessful attempt to ingest the prey, indicating possible intoxication that influences spider mobility (Supplementary Movies 1–3). To find out whether Lyc4 itself or its unknown metabolite(s) caused this spider behaviour, we conducted choice and no-choice assays using irGGPPS- (Fig. 6c–i) and AD-fed (Supplementary Fig. 13d–h) larvae (*n*=21 in choice assays and *n*=30 in no-choice assays) topically coated with water, Lyc4 or RGHGL. More than 90% of these coated compounds could be recovered unmetabolized from the larval body surface after 1 h (*n*=3), demonstrating that the compounds were not degraded during the assay period (Supplementary Fig. 13i). Moreover, no Lyc4 or RGHGL was detected in the washes of water-coated irGGPPS- or AD-fed larvae and also the EV-, irBG1- and irGGPPS-fed larvae that were not coated with any substance, suggesting that these compounds were not externalized by larvae. Prey capture and kill frequency were reduced by at least 80% for Lyc4-coated larvae compared with water- or RGHGL-coated larvae (Fig. 6c–e). Similarly, in choice assays, spiders clearly preferred water- or RGHGL-coated larvae over Lyc4-coated ones, while not showing a clear preference between water- and RGHGL-coated larvae (Fig. 6f–i). Spiders readily captured and killed the water- or RGHGL-coated larvae (Supplementary Movies 4 and 5) but clearly rejected Lyc4-coated larvae after chemosensory assessment (Supplementary Movie 6). In rare incidences during these assays, when spiders tried to ingest the Lyc4-coated larvae, they failed and showed signs of locomotor distress. Together, these results revealed that Lyc4 was the spider deterrent and we infer that the high Lyc4 content of the BG1-silenced larvae hindered spider's prey ingestion and was associated with spider's locomotor distress.

## Discussion

Heiling *et al*.^20^ discovered the occurrence of malonylated HGL-DTGs in *N. attenuata*. They proposed that malonylation facilitates accumulation and distribution of HGL-DTGs in plant and prevents their deglycosylation by plant glycosidases. However, that the malonylation is instantaneously lost in the alkaline larval regurgitant indicates that malonylation's sole role could be *in planta*, for example, to ensure the availability of toxic Lyc4's to the herbivore. Indeed, considering that the lepidopteran herbivore is highly mobile and is likely to devour all parts of the shoot, the concentration and the distribution of Lyc4 could be crucial for the optimization of the plant's defensive strategy^38^. The role of malonylated Lyc4 also appears to be similar to that of phytoanticipins in which, glycosylation ensures the within plant stability of defence molecules by maintaining them in inactive or rather pretoxin states^1^,^2^. However, that the core Lyc4 often occurs in certain tissues of *N. attenuata* in large quantities implies that its stability and toxicity may not be of concern for the plant. In this way, the malonylated Lyc4 could be considered as a type of phytoanticipin, which is decorated for distribution, rather than stability. From *M. sexta*'s perspective, demalonylation appears to be unavoidable due to the need to maintain an alkaline midgut.

As mentioned earlier, the large literature^3^,^11^,^12^ on herbivores' xenobiotic detoxification reveals that glycosylation is an important detoxification mechanism; consequently, the current perception that deglycosylation is often a toxin activation mechanism is widely accepted. On the basis of such literature, we initially hypothesized that Lyc4 is completely deglycosylated by *M. sexta* to release the non-polar toxic 17-HGL, the diterpene backbone of Lyc4; however, this compound was not found in the body, gut contents or frass of larvae. When we identified RGHGL from the frass of Lyc4-fed larvae, we hypothesized that RGHGL was the activated toxin. High incidences of moulting impairment and mortality in larvae feeding on irBG1 foliage and the reappearance of these phenotypes in larvae feeding on Lyc4-complemented B × G foliage (Fig. 4c,d) falsified this hypothesis, from which we inferred that Lyc4 was the toxin and RGHGL was actually the detoxified product. While detoxification by deglycosylation is uncommon in insects, it has been frequently observed in phytopathogenic fungi. For example, the fungus *Gaeumannomyces graminis* (Magnaporthaceae) detoxifies the avenicin saponins (triterpenoid glycosides containing three sugars) of oat by BG-catalysed deglucosylation^39^,^40^. Similarly, various fungi detoxify the tomato steroidal glycoalkaloid α-tomatine (containing four sugars) by removing its C-3 oligosaccharide that renders it incapable of binding to 3β-hydroxy sterols, which leads to the formation of membrane pores and cell lysis^41^,^42^. Notably, in all these cases, fungi completely deglycosylate the toxic glycosides; thus, Lyc4's detoxification is an atypical example of ‘detoxification by partial deglycosylation'.

The activity of glycosides can be attributed not only to their aglycones but also to sugar moieties, especially when compounds contain multiple sugars. Such activity mainly depends on the binding specificity of sugars to the target molecules and receptors. For example, aureolic acid group antibiotics bind to DNA and inhibit the DNA-dependent RNA polymerase; aureolic acid variants differing in their sugar moieties show varying degree of inhibitory activities^43^, but the aglycone is completely unable to bind to DNA^44^. Similarly, a nonsugar sweetener, glycyrrhizic acid (ammonium salt), tastes sweeter if its second glucuronic acid is replaced by glucose or xylose, owing to the differential binding capacity of sugars to the receptors in the taste buds^45^. It is known that binding of glycosides' specific sugars to receptors on the cell surfaces facilitates their active uptake^46^. Conspicuously, higher amounts of Lyc4 than RGHGL in most of the larval tissues (in EV- as well as irBG1-fed larvae) may result from this sugar-specific uptake phenomenon, wherein the uptake of Lyc4 in various tissues is facilitated by the receptor-binding capacity of C-17 glucose, a structural feature absent in RGHGL.

The fact that spider deterrence is caused by Lyc4 and not by RGHGL is consistent with our inference that Lyc4 is the toxin and RGHGL is its detoxified product. That the spiders completely ingest EV- and irGGPPS-fed larvae suggests that *M. sexta* larvae have not evolved sequestration of Lyc4 for their own defence. Spiders' chemosensory-endowed hair on legs and palps can detect the Lyc4 coated on the larval surface. But the spiders are neither deterred by the control and nor by the BG1-silenced larvae, which have ingested Lyc4, mainly because the larvae do not externalize Lyc4. Moreover, the concentrations of Lyc4 in haemolymph and fat body, the tissues in which insects usually sequester the xenobiotics^36^,^47^, were much lower than those found in the midgut suggesting that Lyc4 is not sequestered. Avoiding self-intoxication could be the reason behind not co-opting Lyc4. However, it would be interesting to study the generalist herbivores of *Nicotiana* spp. to evaluate if HGL-DTG co-option has evolved in them; such comparisons between the nicotine metabolism of *Manduca* spp. and generalist herbivores were highly useful in evaluating the costs and benefits of detoxification and co-option of nicotine^37^. The observation that spiders that ingest BG1-silenced larvae show signs of locomotor distress suggests that Lyc4 affects spider's nervous system.

Together, this work reveals that deglycosylation can bring about detoxification and suggests that much remains to be revealed about the detoxification of multiply glycosylated compounds by herbivores and roles of such compounds in tritrophic interactions. It suggests that multiply glycosylated as well as malonylated plant metabolites could be much more useful in pest control than previously thought; consequently, the newly discovered role of insect β-glycosidases in detoxification suggests that these enzymes could be valuable targets for pest control. It demonstrates value of PMRi in molecular ecological research, but given the simplicity and robustness of this trophically mediated gene-silencing technique, it could also become a valuable means of controlling pests in agriculture. The biochemical basis of partial deglycosylations of Lyc4 by BG1 or other β-glycosidases and the mode of action of Lyc4 are interesting future perspectives of this work.

## Methods

### *M. sexta*

*M. sexta* eggs were obtained from an in-house colony in which insects are reared in a growth chamber (Snijders Scientific) at 26 °C:16-h light, 24 °C:8-h dark and 65% relative humidity, until hatching. *M. sexta* eggs used in all the field experiments were kindly provided by Carol Miles (Department of Biological Sciences, Binghamton University, Binghamton, NY). Neonates were fed on various *N. attenuata* lines (EV, irBG1, irGGPPS or B × G) until they were used in glasshouse experiments. In some experiments, neonates were fed on AD^48^ containing 6 mM Lyc4 (aqueous; in 100 μl g^−1^ AD), 6 mM RGHGL (aqueous; in 100 μl g^−1^ AD) or equivalent amount of water (control), to rear the larvae free from any influence of the hostplant.

### Plant material

The 30th generation seeds of an inbred line of *N. attenuata*, which were originally harvested in 1988 from a native population in Utah (USA), were used for the development of stable transgenic lines^49^. Seeds were germinated and plants were grown as explained previously^24^.

Field experiments were conducted at Lytle Ranch Preserve in Santa Clara, Utah, 84765 (37° 08′ 45′′ N, 114° 01′ 11′′ W) in 2013. Seeds of *N. attenuata* EV, irGGPPS and irBG1 lines were imported and released in accordance with Animal and Plant Health Inspection Service notifications (Supplementary Table 3). Planting of transgenic lines in the field plot was performed as described by Kessler *et al*.^50^

### Selection of *glycoside hydrolase* gene sequences for PMRi

The complete coding sequences of 12 *M. sexta glycoside hydrolases* were retrieved from NCBI (accession numbers are given in Supplementary Fig. 9). Their open-reading frames were aligned and the phylogenetic tree was constructed with maximum parsimony method using Clustal W, with 1,000 bootstrapping trials to identify the formation of clusters. Three *BG* sequences (*BG1*, *BG2* and *BG3*) were selected for the (quantitative PCR with reverse transcription) qRT–PCR-based determination of their transcript levels in larval midgut in response to Lyc4 and RGHGL ingestions. A 301-bp cDNA stretch of *BG1*, which was upregulated in response to Lyc4 ingestion, was selected for cloning into a PMRi vector as previously described^24^.

### Plant transformation and Southern hybridization

Stable transgenic PMRi plants were generated by *A. tumefaciens*-mediated transformation of recombinant pSOL8 vector^49^. The pSOL8 vector contained an ir of 301-bp stretch (+84 to +384) of *MsBG1* separated by the *pdk i3* intron and a selectable marker, hygromycin phosphotransferase (*hptII*), for hygromycin resistance^51^. Transgenic lines were screened as described by Gase *et al*.^52^ Single transgene insertions in two independently transformed homozygous irBG1 lines (375-10 and 379-8) were confirmed using Southern hybridization. For Southern hybridization, genomic DNA was extracted from the rosette leaves of untransformed WT and irBG1 plants (line no. 375-10 and 379-8) using a modified cetyltrimethylammonium bromide method as described by Rogers and Bendich^53^. The genomic DNA (10 μg) was completely digested with HindIII and EcoRV, separately. The digested DNA was separated on 1% (w/v) agarose gel and blotted onto a nylon membrane (GeneScreen Plus; Perkin-Elmer) by capillary transfer. Hybridization and detection of the transgene copy number were performed as described by Gase *et al*.^52^

Previously characterized *GGPPS* (NCBI accession no. EF382626)-silenced irGGPPS line of *N. attenuata*^20^ was used as a Lyc4-depleted control to feed *M. sexta* larvae. An EV-transformed *N. attenuata* transgenic line (A-04-266-3) was used as a Lyc4-replete control^28^.

### Harvesting larval tissues

Fourth-instar Larvae were immobilized by placing them on ice before dissection. Haemolymph was collected by clipping the larval horn just before dissection, as described by Kumar *et al*.^24^. Then, larvae were dissected in 0.15 M NaCl to collect malpighian tubules, fat body with skin, foregut, midgut and hindgut. Peritrophic membrane and gut contents were removed before midgut collection. Dissected gut tissues were carefully washed in 0.15 M NaCl to remove any adhering plant material. All the collected tissues were flash-frozen in liquid nitrogen and stored at −80 °C until further use^24^. Lyc4 and RGHGL contents of the entire larval body (without gut contents) were also analysed. For this, all the haemolymph was first collected by opening the larval body with a longitudinal cut on the dorsal skin; then, the gut contents were removed by dissecting the gut and all tissues, together with previously collected haemolymph, were stored for further analyses.

### Collection of larval regurgitant

The regurgitant from fourth-instar *M. sexta* larvae was collected with the help of Teflon tubing connected to a vacuum, after gently agitating the larvae^25^. Collected regurgitant was stored under argon at −80 °C until use.

### Low-molecular-weight RNA isolation and Northern hybridization

Low-molecular-weight RNA was separated from total RNA isolated from larval midgut as well as from leaves using polyethylene glycol (10%) precipitation in the presence of 1 M NaCl^24^.

A 120-bp stretch of the 301-bp-*Ms*BG1 fragment cloned for PMRi was PCR amplified using BG1.3 gene-specific primers (Supplementary Table 2) and used as a template in the labelling reaction. Ten nanogram of PCR product was labelled with α-^32^P isotope using the Rediprime II DNA labelling system (Amersham Biosciences). Low-molecular-weight RNA blotting, hybridization and screening of the blot performed^24^.

### RNA isolation and qRT–PCR

Larval foregut, midgut, hindgut, haemolymph, malpighian tubules and fat body along with the skin were collected from the fourth-instar *M. sexta* larvae and stored in TRI reagent (Invitrogen) at 4 °C. Total RNA was isolated from the stored tissues according to the manufacturer's protocol and was subjected to TURBO DNase (Ambion) treatment to eliminate genomic DNA contamination. Transcript levels of *BG*s were determined by qRT–PCR conducted in Mx3005P Multiplex qPCR system (Stratagene) using qRT–PCR SYBR Green I kit (Eurogentec)^24^. Relative quantification of transcripts was carried out by the comparative D cycle threshold method. *Ms*Ubiquitin levels were taken as internal controls to normalize the abundance of BG transcripts. All the primer pairs (Supplementary Table 2) used in qRT–PCR were designed using Primer3 software version 4.0 (http://primer3.ut.ee/).

### Extraction and GC–MS analysis of 17-HGL (aglycone)

Foregut, midgut, hindgut, haemolymph, fat body along with the skin and frass of EV-fed larvae were used to determine whether *M. sexta* larvae deglycosylated Lyc4 to its aglycone, 17-HGL; EV leaf was also analysed to determine whether the plant glucosidases deglucosylate the HGL-DTGs when herbivores ingest the leaf. Samples of each tissue (100 mg) were homogenized in 1 ml *tert*-butyl methyl ether and then centrifuged at 13.4*g* for 10 min. The supernatant was dehydrated using anhydrous sodium sulphate at room temperature for 30 min. Subsequently, 100 μl of the supernatant was collected in a glass vial and derivatized by adding 10 μl trimethyl sulfonium hydroxide. Derivatized samples were analysed for aglycones using DB5 column in Varian CP-3800 GC-Saturn 4000 ion trap MS^54^,^55^. Leaf tissue was crushed in the alkaline buffer (sodium phosphate buffer, pH-8.5), acidic buffer (sodium phosphate buffer, pH-6) and acidified regurgitant (pH-6), separately, and was incubated at 37 °C for 1 h before extraction.

### Extraction and purification of metabolites from larval frass

Frass of EV-fed *M. sexta* larvae was collected and stored at −80 °C until further use. It was ground in liquid nitrogen and then extracted using 100% methanol (1 ml methanol for 100 mg of frass). Samples were centrifuged at 13.4*g* for 30 min at 4 °C. The supernatants were incubated at −20 °C for 2 h for precipitating proteins and were again centrifuged at 13.4*g* for 30 min at 4 °C and the clear supernatants were collected. Further, the extract was evaporated in a rotary evaporator to one-tenth of its original volume. The concentrated extract was centrifuged at 13.4*g* for 20 min at 4 °C and the supernatant was subjected to fractionation using a reverse-phase HPLC (Luna C-18 (2), 250 × 10 mm; Phenomenex) at a 3-ml min^−1^ flow rate. Two types of solvents (A and B) were used to elute analytes from the column. Solvent A: HPLC grade methanol (Fluka, Germany) and solvent B: millipore water (obtained using a Millipore model Milli-Q Advantage A10). Chromatographic solvent gradients were, from 0 to 20 min (15% of B) and from 20 to 40 min (100% of B). Lyc4 and RGHGL were collected from fractions 46 (23 min) and 48 (24 min), respectively. Collected fractions were dried completely in a vacuum concentrator (3.7 mbar; Concentrator 5301; Eppendorf), and thus obtained pure metabolites were used for further experiments.

### Extraction and U(H)PLC/ESI-QTOF-MS analysis of HGL-DTGs

For analysing Lyc4 and its malonylated forms of Lyc4 in the leaf, larval tissues and larval frass, samples were homogenized and extracted in an acidic extraction buffer A (60% solution 1 (2.3 ml l^−1^ of acetic acid, 3.41 g l^−1^ ammonium acetate, pH-4.8 using 1 M NH_4_OH) and 40% methanol). An internal standard rebaudioside A was always spiked into extraction buffer A (10 ng μl^−1^) before extraction. A amount of 100 mg of homogenized sample was extracted in 1 ml of buffer A. Extracted samples were diluted 1:50 with buffer B (one part of buffer A and nine parts of 40% methanol) and analysed using a U(H)PLC/ESI-QTOF-MS^20^. Retention times and molecular ions of Lyc4, RGHGL and rebaudioside A were determined by loading 1 ng of each of these compounds onto a Fisher Scientific Acclaim C18 (2.1 × 150 mm) U(H)PLC column (particle size 2.2 μM) with solvent A (0.1% acetonitrile, 0.05% formic acid (vol/vol) in ultrapure Millipore water) and solvent B (0.05% formic acid in acetonitrile). The gradient of 0 min 10% B, 1 min 10% B, 9 min 80% B, 10 min 80% B, with a flow rate of 0.3 ml min^−1^ was used. Compounds were detected using a qToF-mass spectrometer (micro TOF QII Bruker Daltonik, Germany) equipped with an ESI source in positive ion mode (instrument settings: capillary voltage, 4,500 V; capillary exit, 130 V; dry gas temperature, 200 °C; dry gas flow, 8 l min^−1^). Calibration was performed using sodium formate clusters (10 mM solution of NaOH in 50/50% (vol/vol) isopropanol/water containing 0.2% formic acid (vol/vol)).

### NMR analysis

^1^H NMR, ^13^C NMR, ^1^H–^1^H COSY, ROESY, TOCSY, HSQC and HMBC experiments were measured on a Bruker Avance 500 NMR spectrometer (Bruker Biospin, Germany), operating at 500.13 MHz for ^1^H and 125.75 MHz for ^13^C. Capillary tubes (2-mm-internal diameter) were used to measure spectra in a TCI cryoprobe (5 mm) at a probe temperature of 300 K. Samples were prepared in methanol-*d*_4_ solvent (85 μl). ^1^H and ^13^C chemical shifts were referenced to the residual solvent signals of methanol-*d*_4_ at δ 3.31 and δ 49.15, respectively.

### Extraction and HPLC analysis of Lyc4 and other metabolites

Stored leaf and frass samples were homogenized in liquid nitrogen using pre-chilled mortar and pestle. Extraction buffer A (1 ml; 60% methanol containing 0.05% glacial acetic acid) and ceramic beads (0.9 g: Sili GmbH, Germany) were added to each aliquot (100 mg) sample, which was again homogenized using Geno/Grinder 2000 (Spex, UK) for 2 min with 600 strokes per min. Homogenized samples were centrifuged at 13.4*g* for 20 min, at 4 °C. The supernatants were recentrifuged at 13.4*g* for 20 min at 4 °C for separating undissolved particles. Clear supernatants were analysed using HPLC (Agilent 1100 series)^29^. Lyc4 from leaf and Lyc4 and RGHGL from frass were quantified using rebaudioside A (Sigma, Germany) as an external standard.

The linearity of the extraction of Lyc4 and RGHGL from frass was determined as follows. Crushed dried frass (100 mg) of irGGPPS-fed larvae was spiked with 2, 4, 6, 8, 16 and 32 μg of each compound. Spiked frass samples were extracted and analysed as mentioned above. The detected quantity of the recovered compound was calculated relative to rebaudioside A. Linearity was determined using the standard curve method, as previously described^32^.

Nicotine, chlorogenic acid, caffeoyl putrescine and rutin were also extracted from the EV, irBG1 (375-10) and irBG1 (379-8) rosette leaves using this method and were analysed by HPLC (Agilent 1100 series) as described by Keinaenen *et al*.^29^

### *In vitro* demalonylation assay

Leaf discs (10 mm) from fully expanded node-2 rosette leaf^56^ of EV plants were collected in liquid nitrogen and homogenized using Dounce homogenizers. A amount of 50 mg of leaf material was used in each assay and incubated with 100 μl of water (control), regurgitant, boiled regurgitant, regurgitant+pronase E (4 units per microlitre of regurgitant; Sigma), regurgitant+inactivated pronase E (heated at 85 °C for 20 min), alkaline buffer (sodium phosphate buffer, pH-8.5), acidic buffer (sodium phosphate buffer, pH-6) and acidified regurgitant (pH-6) in separate treatments at 37 °C for 1 h. Regurgitant was boiled or treated with pronase E to inactivate enzymes in it. During the assay period, assay contents were mixed intermittently. After 1 h, metabolites were extracted and analysed for Lyc4 and its malonylated forms using HPLC.

### *In vitro* BG enzyme assay

Midguts of larvae feeding on EV and irBG1 plants were homogenized using Dounce homogenizers and incubated with either 1 mM of Lyc4 or 1 mM of Lyc4+ 1 mM δ-gluconolactone (BG inhibitor; Sigma, Germany)^57^, for 1 h. Then, the assay contents were extracted and their Lyc4 and RGHGL concentrations were determined using U(H)PLC/ESI-QTOF-MS.

### Excretion efficiency determination assays

Ingestion of Lyc4 and excretion of Lyc4 and RGHGL by *M. sexta* larvae were budgeted using the excretion efficiency determination assays^32^,^33^. Freshly hatched *M. sexta* neonates were allowed to feed on EV and irBG1 rosette-stage plant leaves until they had reached the fourth-instar. The mass of each larva was recorded and then the larvae were starved for 4 h to empty their guts. Larval mass was measured again after starvation. Each larva was then provided with a known mass of leaf material from the same genotype of plant that they had been fed previously. Larvae were then fed for 24 h at 26 °C:16-h light, 24 °C:8-h dark and 60% relative humidity. To absorb the excreted liquids, blotting paper disks of known masses were laid in each assay container. The mass of each larva and that of remaining leaf material were recorded after 24 h feeding. All the larvae were again starved for 4 h to empty their guts. Frass excreted by each larva, during the 24 h of feeding, and the 4 h of starvation were collected, weighed along with the paper disk and stored at −80 °C until further use. Lyc4 levels of leaves of each line were measured by HPLC (Agilent 1100 series). Actual amount of Lyc4 ingested by each larva was calculated based on the amount of foliage ingested. Lyc4 and RGHGL levels in the collected frass samples (along with the blotting paper discs) were also measured and corrected for the mass of the blotting paper disk. The percentage of Lyc4 or RGHGL excreted was determined by calculating the ratio of the amount of compound excreted/amount of Lyc4 in the ingested food × 100.

### Generation of irBG1 × irGGPPS (B × G) plants

Lyc4-depleted and BG1 dsRNA-producing plants were generated by crossing irBG1 (375-10) with irGGPPS (230-5) plants. Crossing was performed by pollinating the stigmas of atherectomized (before pollen maturation) irBG1 flowers with irGGPPS pollen. B × G seeds were collected and germinated. Lyc4 was extracted from the rosette B × G leaves and was analysed using HPLC (Agilent 1100 series). Genomic DNA was isolated from B × G leaves and was subjected to PCR using BG1 primers (Supplementary Table 2) to confirm the presence of *Ms*BG1 cDNA fragment inherited from the irBG1 parent.

### Moulting impairment and mortality analyses

To determine the effect of Lyc4 detoxification suppression on larvae, we carefully monitored the larvae feeding on EV and irBG1 plants for 12 days (until they reached fourth-instar). After moulting impairment and mortality were observed in irBG1-feeding larvae during their each instar change, to determine whether *BG1* silencing or suppressed Lyc4 deglucosylation caused these phenotypes, we used the complementation method. Detached fully expanded rosette leaves of irGGPPS and B × G were coated with Lyc4 and RGHGL (separately; final concentration 6 mM; 4,656 μg Lyc4 and 3,684 μg RGHGL in 100 μl water per gram leaf, respectively) and EV, irBG1, irGGPPS and B × G leaves were coated with 100 μl water (control) using a paint brush. Freshly hatched neonates were reared on these leaves for 12 days (until they reached fourth-instar). Fresh leaf material (Lyc4, RGHGL or water coated) was daily provided to the larvae. Larvae were daily monitored for moulting impairment and mortality. After 12 days, surviving larvae from both control and treatments were used to quantify BG1 transcripts in their midguts by qRT–PCR and Lyc4 and RGHGL levels in their bodies using U(H)PLC/ESI-QTOF-MS.

### Survivorship assays in the field

Diurnal and nocturnal survivorship assays were conducted as described by Kumar *et al*.^32^. *M. sexta* neonates were fed on EV, irBG1 and irGGPPS plants until they reached second-instar. Thirty-two larvae from each *N. attenuata* line were placed on respective rosette-stage plants that were planted across a predator-teemed field in a random spatial array^32^. To analyse diurnal survivorship, larvae were placed on plants in the field at 0600 hours and the number of larvae surviving on each plant was counted at 2000 hours; to analyse nocturnal survivorship, larvae were placed on plants in the field at 2000 hours and surviving larvae were counted at 0600 hours. Moulting-impaired BG1-silenced larvae were not used in these assays.

### Spider predation bioassays

All choice and no-choice assays were conducted for 1 h in a polypropylene container (60 cc), as described by Kumar *et al*.^32^ *C. parallela* spiders collected from in and around the *N. attenuata* field were used as predators in all assays. Spiders were starved for 12 h before conducting assays. Spiders were not reused in these assays. *M. sexta* neonates were fed with their respective experimental diets until they reached late second-instar. Late second-instar *M. sexta* larvae were used as prey in all assays. During the assay, only if the spider captured and killed a larva it was recorded as a spider's choice in a choice assay or as a spider's predation in a no-choice assay. Ingestion of larva was recorded only if the spider consumed an entire larva during the assay.

### No-choice assay

In these assays, only one larva was given to a spider in each assay container. Assays with test and respective control larvae were always performed simultaneously. Whether spiders were the predators that left the killed larvae on irBG1 plants was tested using no-choice assays. Percentages of larvae captured and killed by spiders and larvae ingested (of total captured) by spiders in 1 h were calculated for EV-, irBG1- and irGGPPS-fed larvae. To determine whether Lyc4 or RGHGL content of BG1-silenced larvae influenced spider's prey ingestion behaviour, larvae fed on water-coated EV, irBG1, irGGPPS or B × G leaves, Lyc4-coated irGGPPS or B × G leaves or RGHGL-coated irGGPPS or B × G leaves were used. In the assays conducted to evaluate whether externalized Lyc4 or RGHGL can influence spider's predation behaviour, water-Lyc4- and RGHGL-coated AD-fed larvae were used; AD-fed larvae were used instead of irGGPPS-fed larvae to remove the effect of nicotine present in irGGPPS plants because *M. sexta* larvae exhale the ingested nicotine to deter spiders^32^.

### Choice assay

In these assays, each spider was allowed to choose between one test and one control larvae. Following combinations of test and control larvae were used in the assays conducted to determine whether spiders can choose between control, Lyc4-depleted and Lyc4 metabolism-suppressed larvae: irBG1 fed+EV fed, irBG1 fed+irGGPPS fed and irGGPPS fed+EV fed. Similarly, the following combinations of test and control larvae were used in the assays conducted to find whether Lyc4 or RGHGL deterred spiders: Lyc4 coated+water coated, RGHGL coated+water coated and Lyc4 coated+RGHGL coated; AD-fed larvae were used in these assays. Each spider was allowed only one choice during the 1-h assay. Spiders' choices of larvae were expressed in terms of the percentage of spiders that chose larvae from each test treatment.

### Coating *M. sexta* larvae with water, Lyc4 or RGHGL

AD-fed *M. sexta* larvae (50±5 mg FM) were coated with 50 μl water (control) or 50 μl of 6 mM aqueous Lyc4 or RGHGL, using a 10-μl-micropipette tip. Coated compounds were allowed to air dry for 5 min, before using the larvae for assays.

### Stability of Lyc4 and RGHGL in frass

To test whether Lyc4 and RGHGL degrade in the excreted frass during the 24 h of excretion efficiency determination assay, these metabolites were separately spiked onto fresh frass (0.5% in 50 mg (FM) frass) and incubated for 24 h at the excretion efficiency determination assay. Samples immediately extracted after spiking (0 h incubated) were used as controls. Metabolites were extracted and quantified from 0 and 24 h samples using HPLC (Agilent 1100 series).

### Stability of Lyc4 and RGHGL in coated leaf and larvae

To test whether Lyc4 and RGHGL coated on leaves degraded during assays, these metabolites were extracted from the coated leaves after 0 h (control) and 24 h (a period for which these leaves were fed to larvae) incubations. Extracted metabolites were analysed using U(H)PLC/ESI-QTOF-MS.

Similarly, to test whether Lyc4 and RGHGL coated on larval body surface degraded during assays, these metabolites were extracted from the coated larvae after 0-h (control) and 1-h (predation assay period) incubations. After 0 and 1 h, larvae were washed in 1 ml buffer A (containing 10 ng μl^−1^ rebaudioside A) and these washes were analysed for metabolites using U(H)PLC/ESI-QTOF-MS.

### Analysis of Lyc4 or RGHGL externalization by larvae

To test whether the larvae externalize Lyc4 or RGHGL, second-instar EV-, irBG1- and irGGPPS-fed and water-coated irGGPPS- and AD-fed larvae (*n*=3) were washed with 1 ml buffer A (containing 10 ng μl^−1^ rebaudioside A) and the washes were analysed using U(H)PLC/ESI-QTOF-MS, as described above.

### Statistical analyses

All the quantitative data (metabolites in leaf material, larval tissues, and larval frass and BG1 transcript levels) were analysed by one-way analysis of variance and the statistical significance (*P*≤0.05) was determined by Fisher's least significant difference or Games Howell *post hoc* test. Mead's resource equation method^58^ was used to determine the adequate sample sizes. Homogeneity of variance of sample groups was tested using Levene's test^59^; sample groups with unequal variances were analysed using Welch's test^60^. Significance (*P*≤0.05) of the binary results of all moulting impairment, mortality, survivorship and predation assays was evaluated using Fisher's exact test. These data were normalized by calculating the percentages for each column to facilitate visual comparisons. Percentages were also analysed by the Fisher's exact test and the significance applicable to both frequencies and percentages are shown in the figures.

## Additional information

**How to cite this article:** Poreddy, S. *et al*. Detoxification of hostplant's chemical defence rather than its anti-predator co-option drives β-glucosidase-mediated lepidopteran counteradaptation. *Nat. Commun*. 6:8525 doi: 10.1038/ncomms9525 (2015).

## Supplementary Material

Supplementary Information Supplementary Figures 1-13 and Supplementary Tables 1-3

Supplementary Movie 1 Spider's prey ingestion behavior when offered with empty vector (EV)-fed Manduca sexta larvae. A spider in the assay container (50cc) was offered M. sexta larvae (second-instar) that had fed since hatching on EV plants. Video (taken after ~15 min of prey capture during the no-choice assays) shows how the spider readily ingests the Lyc4-replete EV-fed larvae.

Supplementary Movie 2 Spider's prey ingestion behavior when offered with inverted repeat geranylgeranyl pyrophosphate synthase (irGGPPS)-fed M. sexta larvae. A spider in the assay container (50cc) was offered M. sexta larvae (second-instar) that had fed since hatching on irGGPPS plants. Video (taken after ~15 min of prey capture during the no-choice assays) shows how the spider readily ingests Lyc4-deplete irGGPPS-fed larvae.

Supplementary Movie 3 Spider's prey ingestion behavior when offered with irBG1-fed M. sexta larvae. A spider in the assay container (50cc) was offered M. sexta larvae (second-instar) that had fed since hatching on irBG1 plants. Video (taken after ~15 min of prey capture during the no-choice assays) shows how the spider fails to ingest Lyc4-replete irBG1-fed larvae (which in turn had silenced their midgut-expressed β-glucosidase 1 transcripts) and shows signs of distress after abandoning the prey. Please note that in the early stages of project, irBG1 line was called as irGLY (as seen in this Movie) before identification of the gene as a BG.

Supplementary Movie 4 Spider's prey capture behavior when offered with artificial diet (AD)-fed M. sexta larvae coated with Lyc4. A spider in the assay container (50cc) was offered M. sexta larvae (second-instar) that had fed since hatching on AD. Video shows how spiders deterred by the AD-fed larvae coated with Lyc4.

Supplementary Movie 5 Spider's prey capture behavior when offered with AD-fed M. sexta larvae coated with water. A spider in the assay container (50cc) was offered M. sexta larvae (second-instar) that had fed since hatching on AD. Video shows how spiders readily capture the AD-fed larvae coated with water.

Supplementary Movie 6 Spider's prey capture behavior when offered with AD-fed M. sexta larvae coated with RGHGL. A spider in the assay container (50cc) was offered M. sexta larvae (second-instar) that had fed since hatching on AD. Video shows how spiders readily capture the AD-fed larvae coated with RGHGL. Please note that in the early stages of project, RGHGL was called as X614 (as seen in this Movie), based on its molecular mass.
