# Supplementary material for: Detoxification of hostplant's chemical defence rather than its anti-predator co-option drives β-glucosidase-mediated lepidopteran counteradaptation
Source: Nat Commun. 2015 Oct 7;6:8525. doi: 10.1038/ncomms9525 (PMC4633822; doi:10.1038/ncomms9525)
Supplement: Supplementary Information — Supplementary Figures 1-13 and Supplementary Tables 1-3 [file ncomms9525-s1.pdf]

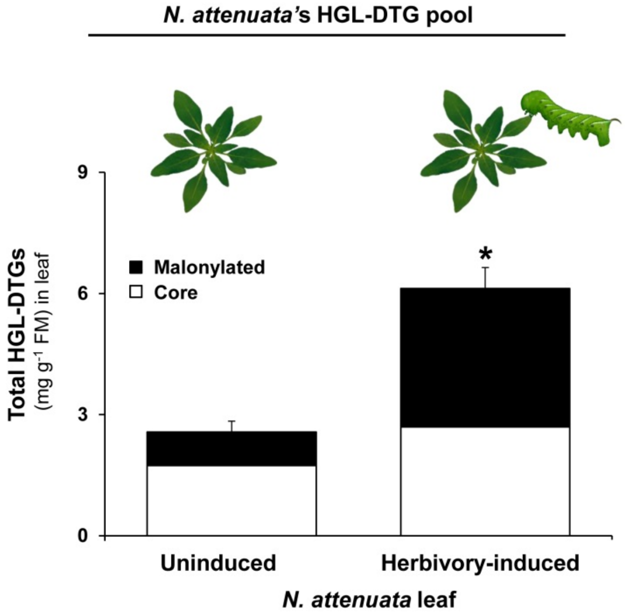

Supplementary Figure 1. HGL-DTG levels in uninduced and herbivory induced leaves. Total HGL-DTG concentrations [$F_{1,4}$= 36.88, $P \leq 0.0037$; significant differences (threshold: $P \leq 0.05$) between means (± SE) determined by Fisher's LSD test (one-way ANOVA); n= 3] and proportions of malonylated and non-malonylated forms in uninduced and *M. sexta* herbivory-induced *N. attenuata* leaves. Together with the data in Fig. 1b, which shows proportions of various HGL-DTGs in uninduced and induced leaves, this data shows the quantitative increase contributed by increases in Lyc4, Nic1 and Nic2 contents in herbivory-induced leaves.

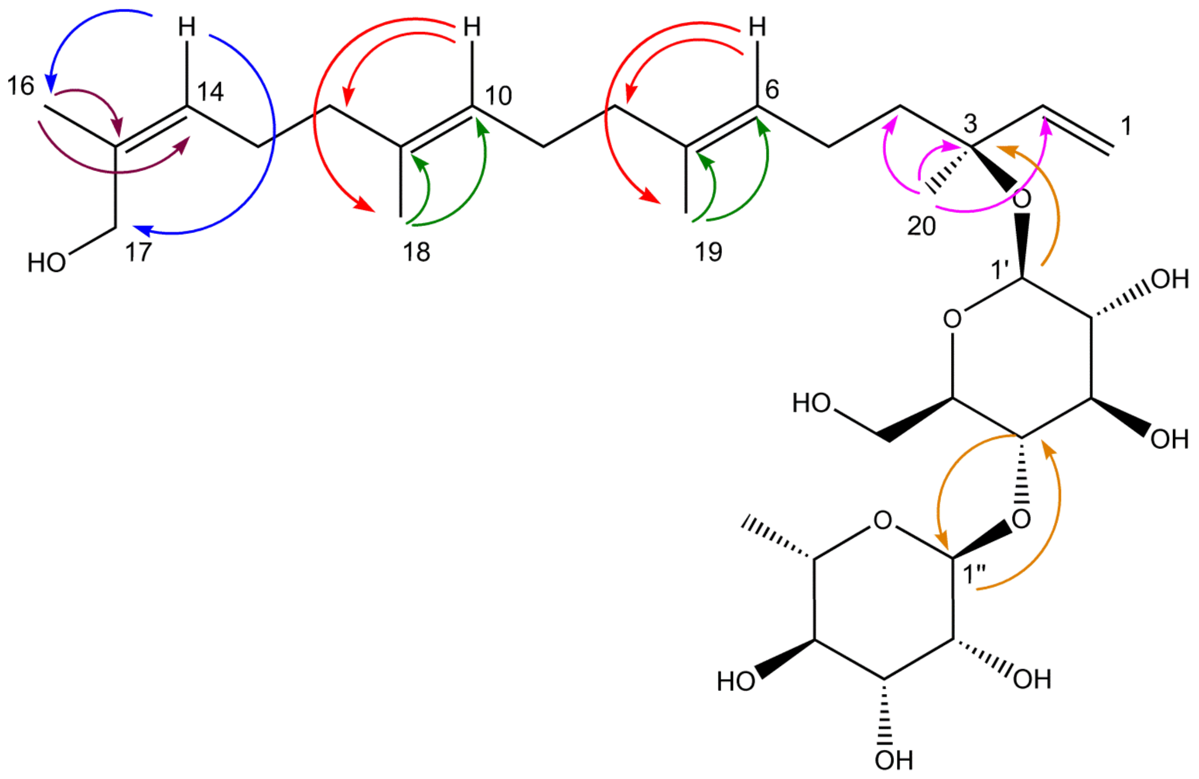

Supplementary Figure 2: Structure and key HMBC correlations ($^{1}H \rightarrow ^{13}C$) of 3-*O*-[α-rhamnopyranosyl-(1→4)-β-glucopyranosyl]-17-hydroxygeranyllinalool (RGHGL). The colors of the arrows correspond to the colors of the cross signals in the spectra shown in Supplementary Fig. 3-7.

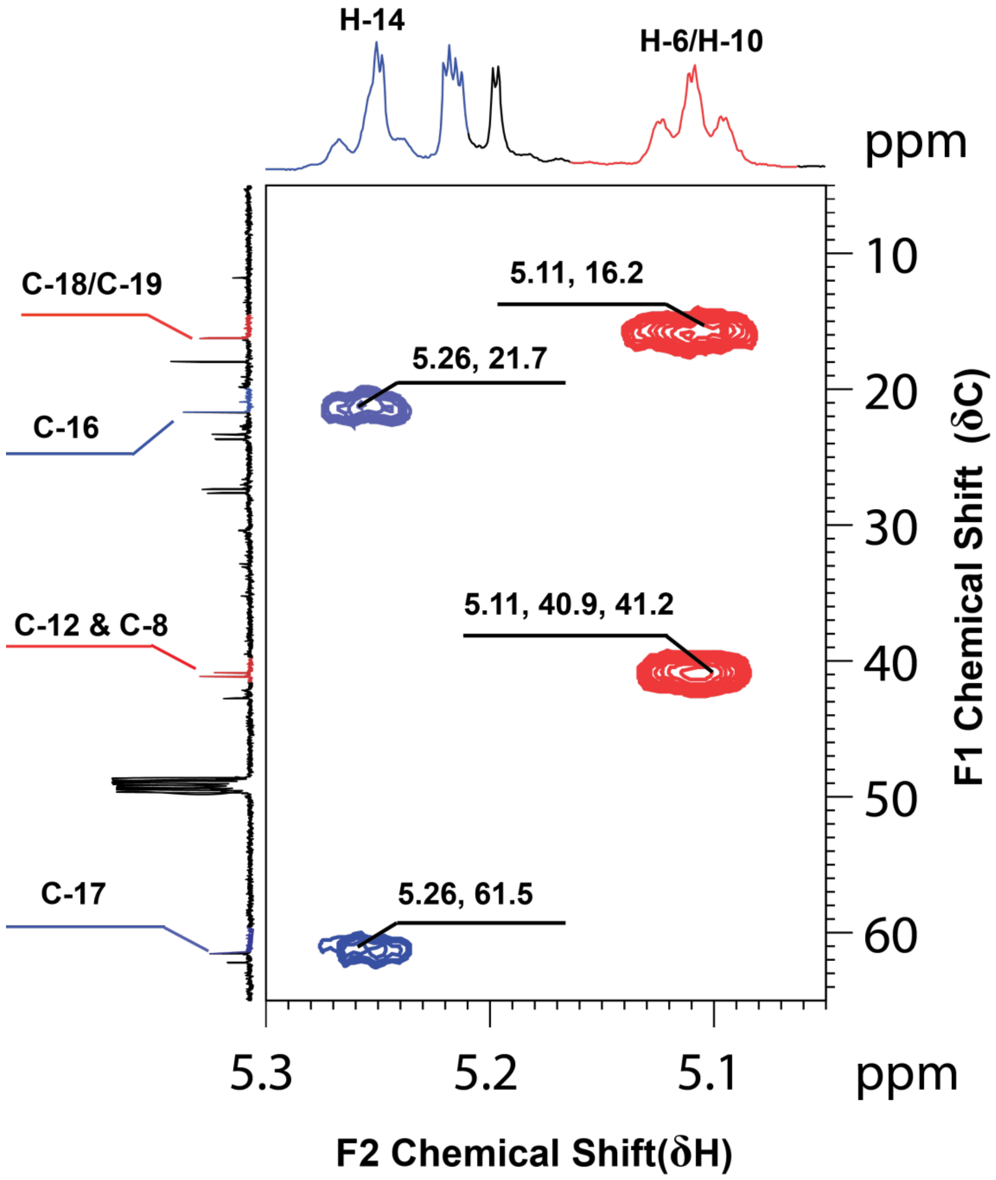

Supplementary Figure 3: Partial HMBC spectrum (500 MHz, MeOH-d_4) of RGHGL

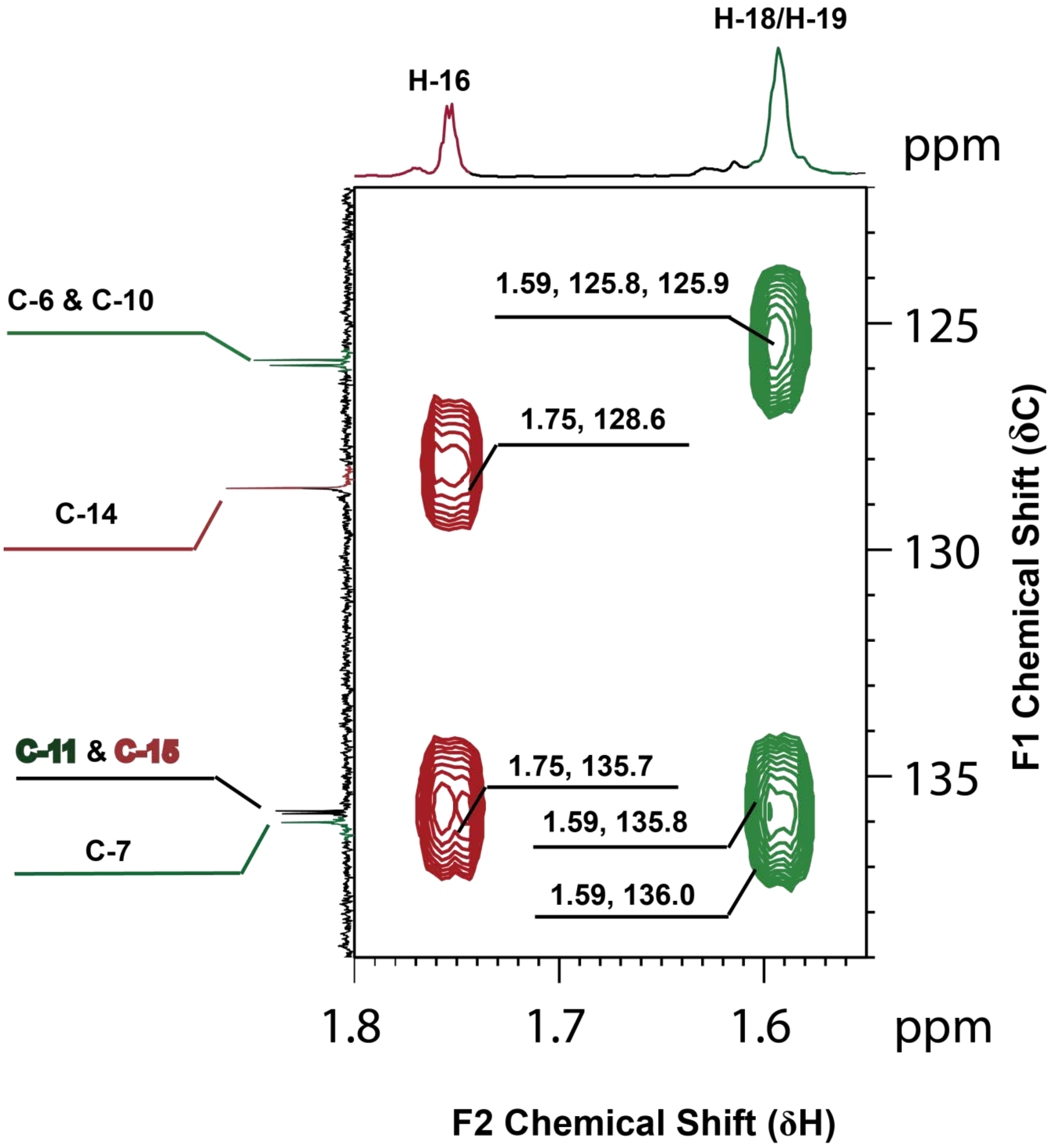

Supplementary Figure 4: Partial HMBC spectrum (500 MHz, MeOH-d_4) of RGHGL

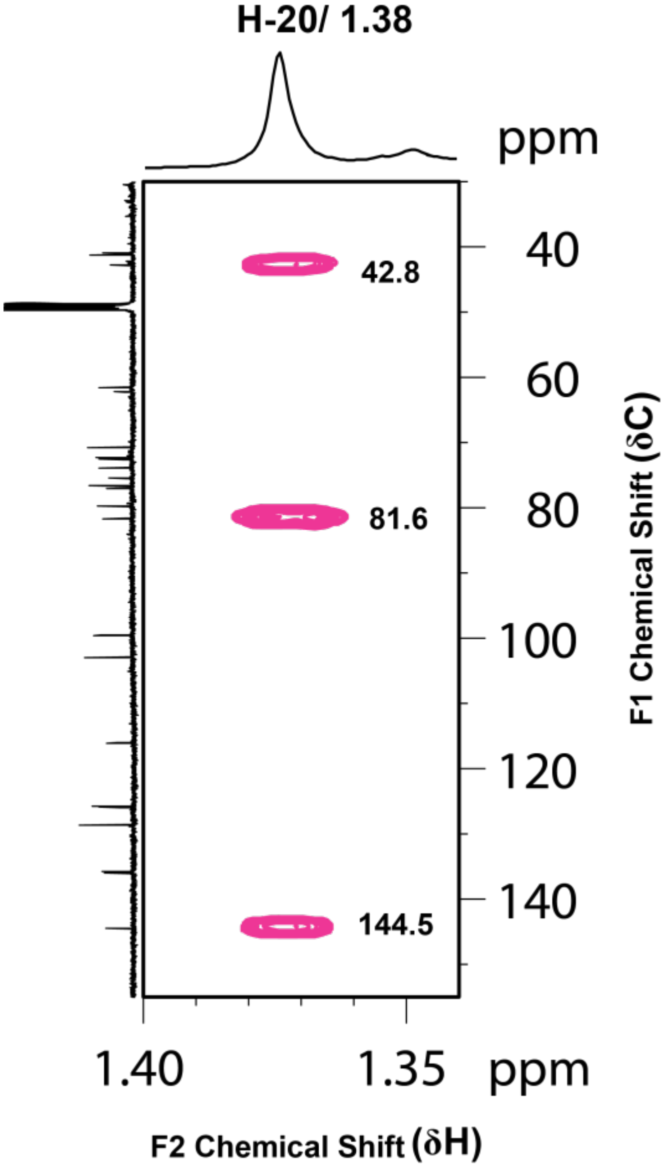

Supplementary Figure 5: Partial HMBC spectrum (500 MHz, MeOH-d_4) of RGHGL

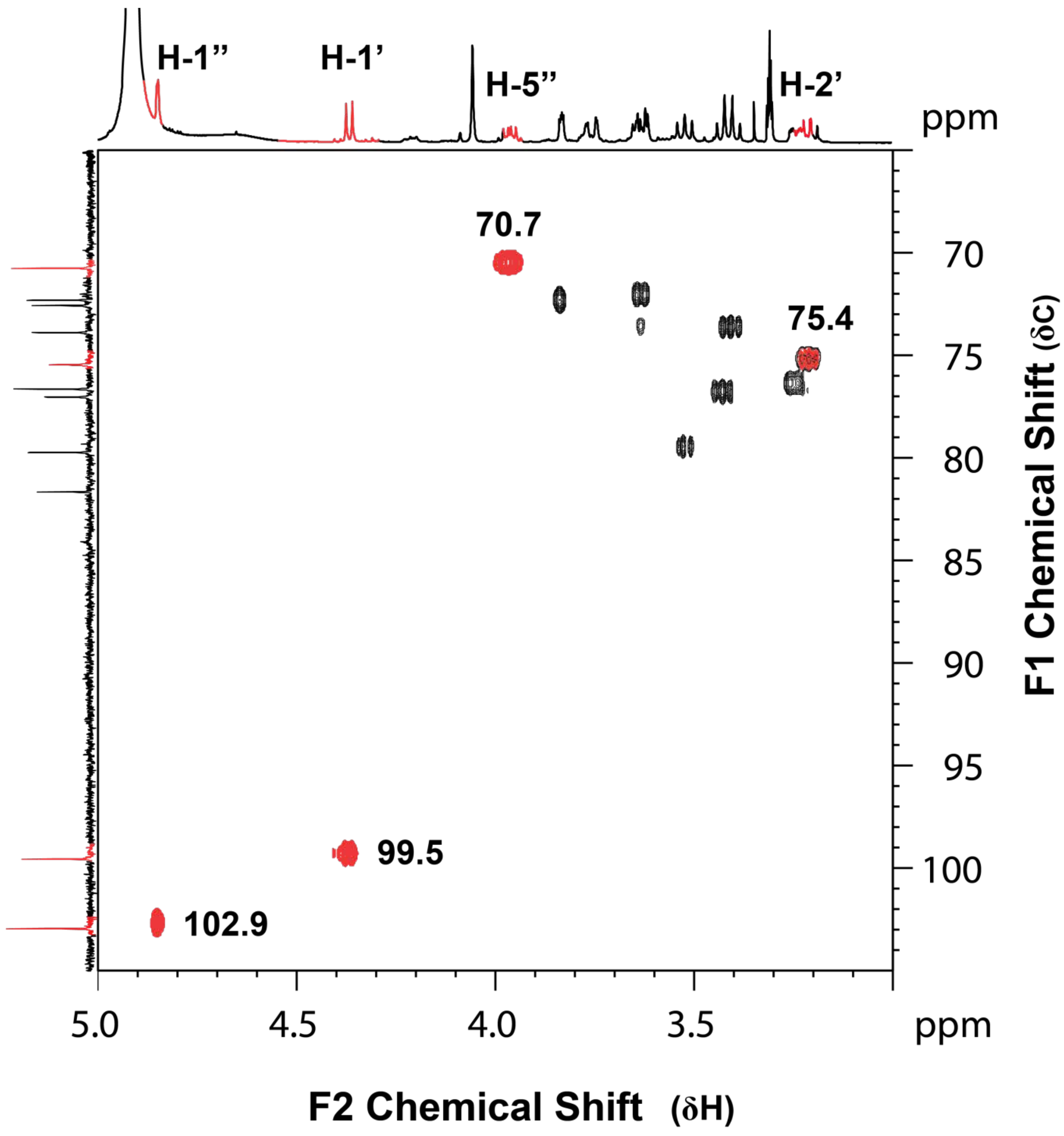

Supplementary Figure 6: Partial HSQC spectrum (500 MHz, MeOH-d_4) of RGHGL

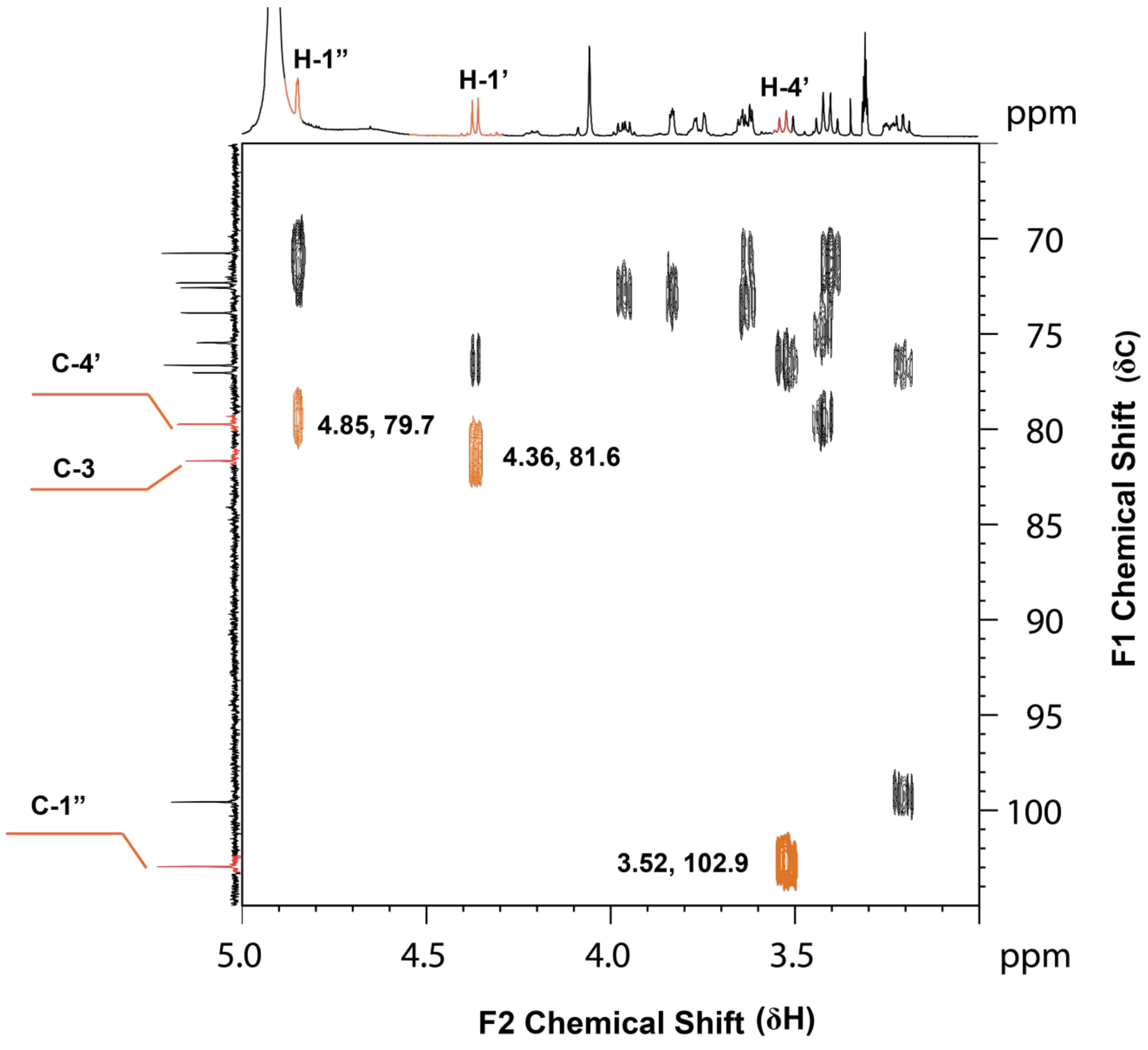

Supplementary Figure 7: Partial HMBC spectrum (500 MHz, MeOH-d_4) of RGHGL

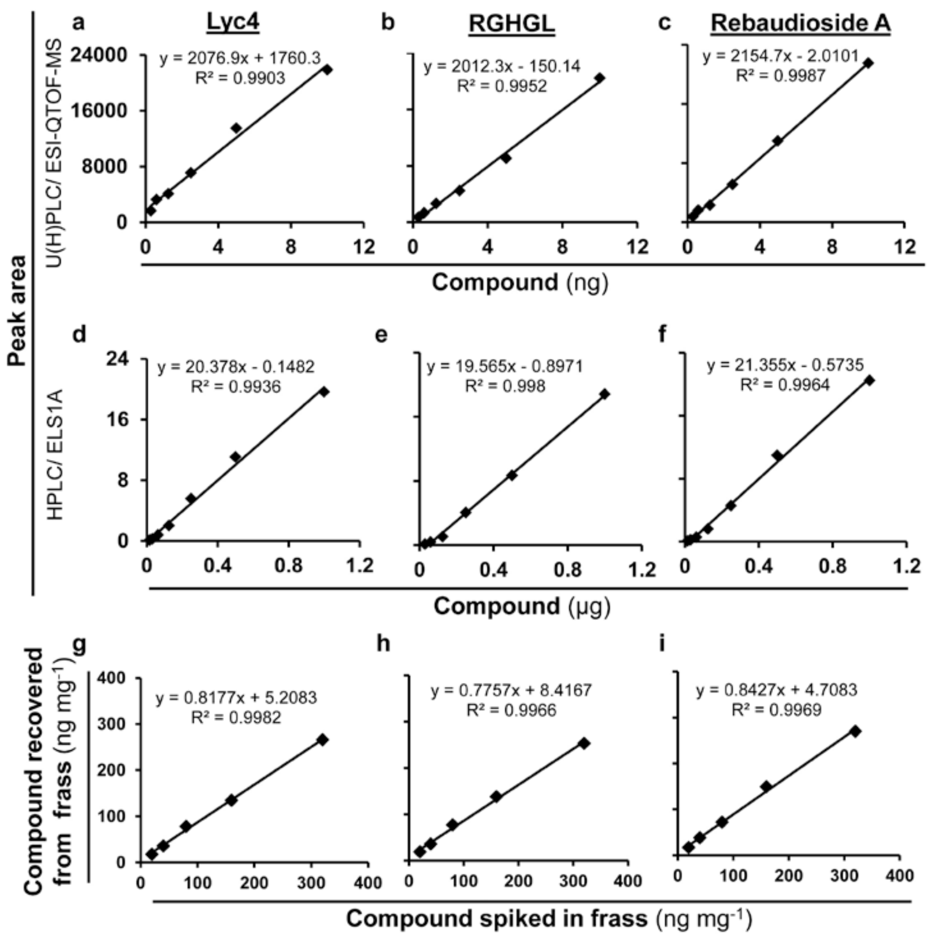

Supplementary Figure 8. U(H)PLC/ ESI-QTOF-MS based analysis of Lyc4, RGHGL and rebaudioside A. Standard curves of (a) Lyc4 (b) RGHGL and (c) rebaudioside A (internal standard) revealed linear responses of U(H)PLC/ESI-QTOF MS to them (n= 3 for each concentration of a compound); limit of detection of rebaudioside A was 0.3 ng. Standard curves of (d) Lyc4 and (e) RGHGL (f) rebaudioside A revealed linear responses of HPLC-ELS1A to them (n= 3 for each concentration of a compound); rebaudioside A was used as an external standard for the relative quantification of these compounds by HPLC-ELS1A and its limit of detection was 15 ng. Plots showing linearity in extraction efficiency of (g) Lyc4 and (h) RGHGL (i) rebaudioside A, from standard addition experiments with frass; for both the compounds, extraction efficiency was >90% when 2, 4, 6, 8, 16 and 32 µg of each compound was spiked to 100 mg frass (n= 3 for each spiking concentration) before extraction and analyzed by HPLC-ELS1A.

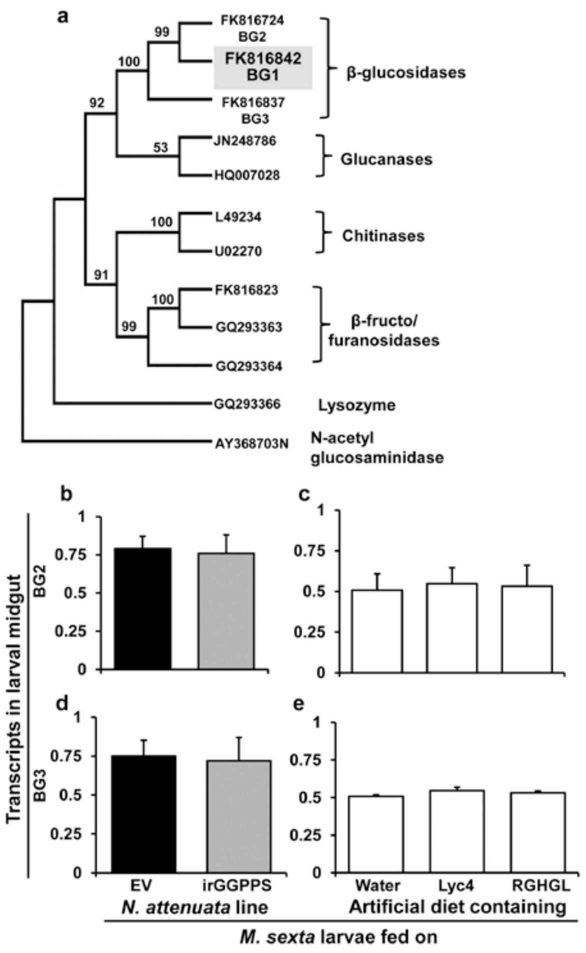

Supplementary Figure 9. ***M. sexta*** **possesses three** ***BG*****s;** ***Ms*****BG2 and** ***Ms*****BG3 transcripts are not upregulated upon Lyc4 ingestion.** (a) Phylogeny of *M. sexta* glycoside hydrolases (only ORFs) deciphered using Clustal-W (thousand bootstrapping trials; only the bootstrap values >50 displayed) showing that *M. sexta* transcriptome contains three *BG*s; *MsBG2* and *MsBG3* are 60.2% and 51.5% similar to *MsBG1*, respectively. BG2 transcripts (relative to ubiquitin) in midguts of fourth-instar larvae feeding on (b) Lyc4-containing EV and Lyc4-deplete irGGPPS plants (n= 6) and (c) artificial diet containing water (control), 6 mM Lyc4 or RGHGL (n= 6). BG3 transcripts (relative to ubiquitin) in midguts of fourth-instar larvae feeding on (d) Lyc4-containing EV and Lyc4-deplete irGGPPS plants (n= 6) and (e) artificial diet containing water (control), 6 mM Lyc4 or RGHGL (n= 6).

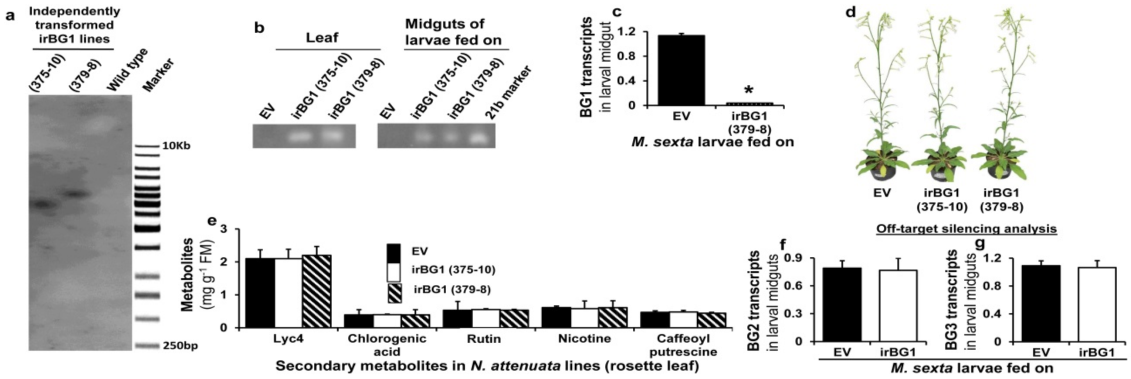

Supplementary Figure 10. Characterization of transgenic irBG1 *N. attenuata* lines and BG1-silenced *M. sexta* larvae. (a) Southern hybridization over the *Hind*III digested genomic DNA, showing the presence of a single copy of transgene fragment inserted in two independently generated transgenic irBG1 *N. attenuata* lines (375-10 and 379-8); wild type control shows the absence of transgene insertion. 1kb DNA ladder was used as a size marker. (b) Northern hybridization showing the presence of BG1 small RNA in irBG1 (375-10) and irBG1 (379-8) *N. attenuata* leaves and in the midguts of fourth-instar larvae feeding on the respective stable transgenic lines; RNA from EV leaves and midgut of larvae those were feeding on EV leaves were used as negative controls, respectively. (c) BG1 transcripts (relative to ubiquitin) in midguts of fourth-instar larvae feeding on the second independently transformed irBG1 line (379-8) [$F_{1,10}$= 940.5, $P \leq 0.0001$; significant differences (threshold: $P \leq 0.05$) between means (± SE) determined by Fisher's LSD test (one-way ANOVA); n= 6], showing equal silencing as that achieved by feeding on irBG1 (375-10) (shown in Fig. 3E). (d) Flowering EV and independently transformed irBG1 plants (375-10 and 379-8) plants showing that the transformation of PMRi construct did not affect plant morphology. (e) Concentrations of various secondary metabolites in leaves of EV and independently transformed irBG1 lines (375-10 and 379-8) (n= 5); since morphologies and the secondary metabolite concentrations and *MsBG1* silencing efficiencies of 375-10 and 379-8 did not differ, 375-10 was randomly selected for the further experimentation. Transcripts (relative to ubiquitin) of (f) *MsBG2* (60.2% similar to *MsBG1*) and (g) *MsBG3* (51.5% similar to *MsBG1*) in the midguts of fourth-instar EV- and irBG1-feeding larvae showing that the off-target silencing of *MsBG2* and *MsBG3* had not occurred while silencing *MsBG1* (n= 6).

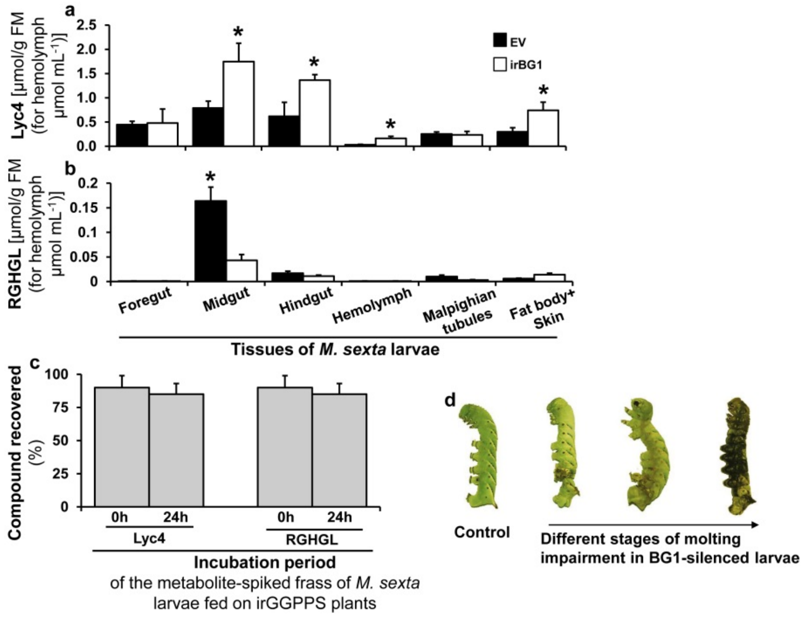

Supplementary Figure 11. Characterization of BG1-silenced *M. sexta* larvae. Concentration of (a) Lyc4 [midgut: $F_{1,11}$= 2.96, $P \leq 0.05$; hindgut: $F_{1,11}$= 6.41, $P \leq 0.02$; hemolymph: $F_{1,8}$= 14.9, $P \leq 0.005$; fat body+ skin: $F_{1,11}$= 5.926, $P \leq 0.03$; significant differences (threshold: $P \leq 0.05$) between means (± SE) determined by Fisher's LSD test (one-way ANOVA, separately conducted for each tissue);] and (b) RGHGL [midgut: $F_{1,11}$= 13.7, $P \leq 0.005$; significant differences (threshold: $P \leq 0.05$) between means (± SE) determined by Fisher's LSD test (one-way ANOVA, separately conducted for each tissue);] in foregut, midgut, hindgut, hemolymph, Malpighian tubules and skin with fat body of fourth-instar larvae feeding on EV and irBG1 (375-10) *N. attenuata* plants. For Lyc4 as well as RGHGL concentrations in foregut, midgut, hindgut, hemolymph, Malpighian tubules and skin with fat body of EV-fed larvae, n= 7, 7, 7, 6, 5 and 7, respectively and for Lyc4 concentration in foregut, midgut, hindgut, hemolymph, Malpighian tubules and skin with fat body of irBG1-fed larvae, n= 7, 6, 6, 4, 5 and 6, respectively. (c) Lyc4 and RGHGL are not degraded in frass over the 24 h period of the excretion efficiency determination assays. Fresh frass was spiked with each metabolite to attain the final concentration of 0.5%; the spiked frass was extracted and analyzed after zero and 24 h of incubation to quantify the recovered metabolite (n= 3). (d) Stages of molting impairment leading to mortality in *M. sexta* larvae feeding on irBG1 plants.

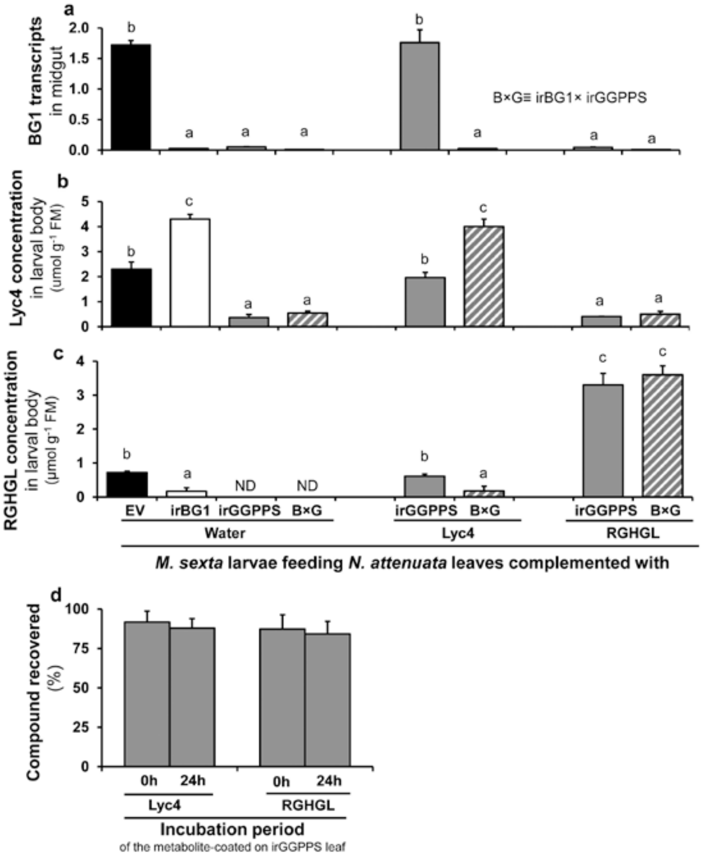

Supplementary Figure 12. BG1 transcripts and Lyc4 and RGHGL concentrations in larvae feeding on Lyc4- and RGHGL-supplemented leaves. (a) BG1 transcripts (relative to ubiquitin) in midguts [$F_{7,40}$= 9253, $P \leq 0.0001$; significant differences (threshold: $P \leq 0.05$) between means (± SE) determined by Games Howell test (Welch's ANOVA); n= 6] and (b) Lyc4 [$F_{7,24}$= 75.39, $P \leq 0.0001$; significant differences (threshold: $P \leq 0.05$) between means (± SE) determined by Games Howell test (Welch's ANOVA); n= 4] and (c) RGHGL [$F_{7,24}$= 50.77, $P \leq 0.0001$; significant differences (threshold: $P \leq 0.05$) between means (± SE) determined by Games Howell test (Welch's ANOVA); n= 4] concentrations in the bodies of *M. sexta* larvae after 8d feeding on water coated EV, irBG1, irGGPPS and B×G leaves, Lyc4 coated (final concentration 6 mM) irGGPPS and B×G leaves and RGHGL coated (final concentration 6 mM) irGGPPS and B×G leaves. (d) Lyc4 and RGHGL coated on irGGPPS leaf are not degraded over the 24 h period. Each detached leaf was coated with Lyc4 or RGHGL to attain the final concentration of 6 mM and was extracted and analyzed after zero and 24 h of incubation to quantify the recovered metabolite (n= 5).

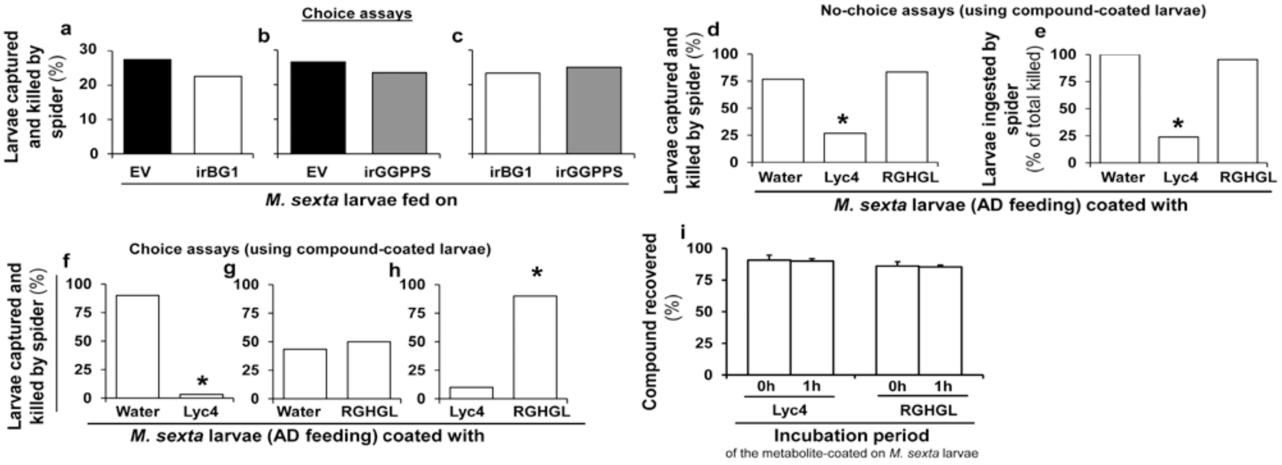

Supplementary Figure 13. Spiders do not choose between EV- irBG1- and irGGPPS-fed larvae. Spider's prey capture and killing (%) in choice assays (1 h) on second-instar *M. sexta* larvae feeding on (a) EV or irBG1, (b) EV or irGGPPS and (c) irBG1 and irGGPPS plants (n= 30 in all assays). (d) Spider's prey capture and killing (%) [significant differences ($P \leq 0.05$) determined by Fisher's exact test of frequencies; n= 30] and (e) prey ingestion (% of total killed) [significant differences ($P \leq 0.05$) determined by Fisher's exact test of frequencies; n= 30] in no-choice assays (1 h) on second-instar water-, Lyc4- or RGHGL-coated (final concentration 6 mM for both Lyc4 and RGHGL) *M. sexta* larvae feeding on AD. Spider's prey capture and killing (%) in choice assays (1 h) on AD-feeding second-instar *M. sexta* larvae coated with (f) water or Lyc4 (final concentration 6 mM) [significant difference ($P \leq 0.05$) determined by Fisher's exact test of frequencies; n= 21], (g) water or RGHGL (final concentration 6 mM) (n= 21) and (h) Lyc4 (final concentration 6 mM) or RGHGL (final concentration 6 mM) [significant difference ($P \leq 0.05$) determined by Fisher's exact test of frequencies; n= 21]. Please note that prey capture and killing percentages using AD-fed larvae are higher than those obtained using irGGPPS-fed larvae (Fig. 6 d-f and h-i) because AD does not contain nicotine, which larvae ingest from irGGPPS plants and exhale to deter spiders. (i) Lyc4 and RGHGL topically coated to the larval body are not degraded over the period of choice or no-choice assays (1 h). Each larva was coated with either Lyc4 or RGHGL to attain the final concentration of 6 mM; it was washed after zero and 1 h of incubation and the wash was analyzed to quantify the recovered metabolite (n= 3).

Supplementary Table 1. ^{1}**H NMR (500 MHz) and** ^{13}**C NMR (125 MHz) chemical shifts for RGHGL in MeOH-*d*$_4$**

Position	δ_H, *multiplet*, *J* (Hz)	δ_C
1a	5.23, *dd*, 17.8, 1.2	116.0
1b	5.20, *dd*, 11.1, 1.2	
2	5.93, *dd*, 17.8, 11.1	144.5
3		81.6
4	1.59, *m*	42.8
5	2.05, *m*	23.7
6	5.11, *t*, 7.3	125.8*
7		136.0
8	1.99, *m*	41.2**
9	2.08, *m*	27.6
10	5.11, *t*, 7.3	125.9*
11		135.8
12	1.99, *m*	40.9**
13	2.15, *m*	27.4
14	5.26, *t*, 7.1	128.6
15		135.7
16	1.75, *s*	21.7
17	4.06, *s*	61.5
18	1.59, *s*	16.2
19	1.59, *s*	16.2
20	1.38, *s*	23.3

Position	δ_H, *multiplet*, *J* (Hz)	δ_C
**Glucose**		
1'	4.36, *d*, 8.0	99.5
2'	3.20, *dd*, 8.0, 9.2	75.4
3'	3.42, *dd*, 9.2, 9.4	77.0
4'	3.52, *dd*, 9.4, 9.4	79.7
5'	3.24, *m*	76.6
6'a	3.76, *dd*, 12.0, 2.2	62.2
6'b	3.64, *dd*, 12.0, 4.3	
**Rhamnose**		
1''	4.85, *d*, 1.7	102.9
2''	3.83, *dd*, 3.3, 1.7	72.5
3''	3.63, *dd*, 9.6, 3.3	72.3
4''	3.40, *dd*, 9.6, 9.4	73.9
5''	3.96, *dq*, 9.4, 6.2	70.7
6''	1.26, *d*, 6.2	18.0

*, **: May be interchanged

Supplementary Table 2. *M. sexta* gene primers used in various experiments

Primer pair No	Gene	Primer sequences (5'-3')	Use
1	*MsBG1.1*	For- CTCGCTTGTTATGGCGGGT<br>Rev- GCGGCAGTGGCTGCAC	Amplification of 301bp fragment to clone in PMRi vector
2	*MsBG1.2*	For- TCGTCCTTCTCGCTTGTTATG<br>Rev- GCTGCACCAAACAGAAATCC	Transcript quantification and testing the silencing efficiency of *M. sexta* BG1
3	*MsBG1.3*	For- TCGAGTCCTAGCTCTCTCATCA<br>Rev- GGACAAAACGTGTCACATGGTA	Generation of probe for northern hybridization
4	*MsBG2*	For- CCACGGTCACAAGTTAAGGC<br>Rev- CTTTTGCCGTCCTCATTCCAC	Transcript quantification and testing the co-silencing efficiency of *M. sexta* BG2
5	*MsBG3*	For- GGATCTGCCGCAAAGACTG<br>Rev- TCACTCTATCGCCGAAGTTCTC	Transcript quantification and testing the co-silencing efficiency of *M. sexta* BG3

Supplementary Table 3. APHIS notification numbers for importing transgenic *N. attenuata* seeds and releasing plants at the field station, Utah, USA.

Line	Import #	Year	Release#
EV	07-341-101n	2013	13-051-101r
irGGPPS	07-341-101n	2013	13-051-101r
(*NaGGPPS* NCBI accession no. EF382626)			
irBG1	10-004-105m	2013	13-051-101r
(*MsBG1* NCBI accession no. FK816842)			
